# From fossils to mind

**DOI:** 10.1038/s42003-023-04803-4

**Published:** 2023-06-13

**Authors:** Alexandra A. de Sousa, Amélie Beaudet, Tanya Calvey, Ameline Bardo, Julien Benoit, Christine J. Charvet, Colette Dehay, Aida Gómez-Robles, Philipp Gunz, Katja Heuer, Martijn P. van den Heuvel, Shawn Hurst, Pascaline Lauters, Denné Reed, Mathilde Salagnon, Chet C. Sherwood, Felix Ströckens, Mirriam Tawane, Orlin S. Todorov, Roberto Toro, Yongbin Wei

**Affiliations:** 1 Bath Spa University, Bath, UK; 2 Laboratoire de Paléontologie, Évolution, Paléoécosystèmes et Paléoprimatologie (PALEVOPRIM), UMR 7262 CNRS & Université de Poitiers, Poitiers, France; 3 University of Cambridge, Cambridge, UK; 4 Division of Clinical Anatomy and Biological Anthropology, University of Cape Town, Cape Town, South Africa; 5 UMR 7194, CNRS-MNHN, Département Homme et Environnement, Musée de l’Homme, Paris, France; 6 Skeletal Biology Research Centre, School of Anthropology and Conservation, University of Kent, Canterbury, UK; 7 Evolutionary Studies Institute, University of the Witwatersrand, Johannesburg, South Africa; 8 Department of Anatomy, Physiology and Pharmacology, College of Veterinary Medicine, Auburn University, Auburn, AL USA; 9 University of Lyon, Université Claude Bernard Lyon 1, Inserm, Stem Cell and Brain Research Institute U1208, F-69500 Bron, France; 10 Department of Anthropology, University College London, London, UK; 11 Department of Human Origins, Max Planck Institute for Evolutionary Anthropology, Deutscher Platz 6, D-04103 Leipzig, Germany; 12 Institut Pasteur, Université Paris Cité, Unité de Neuroanatomie Appliquée et Théorique, F-75015 Paris, France; 13 Department of Complex Trait Genetics, Vrije Universiteit Amsterdam, Amsterdam, The Netherlands; 14 University of Indianapolis, Indianapolis, IN USA; 15 Institut royal des Sciences naturelles, Direction Opérationnelle Terre et Histoire de la Vie, Brussels, Belgium; 16 Department of Anthropology, University of Texas at Austin, Austin, TX USA; 17 CNRS, CEA, IMN, GIN, UMR 5293, Université Bordeaux, Bordeaux, France; 18 PACEA UMR 5199, CNRS, Université Bordeaux, Pessac, France; 19 Department of Anthropology, The George Washington University, Washington, DC USA; 20 C. & O. Vogt Institute for Brain Research, University Hospital Düsseldorf, Heinrich-Heine University Düsseldorf, Düsseldorf, Germany; 21 Ditsong National Museum of Natural History, Pretoria, South Africa; 22 School of Natural Sciences, Macquarie University, Sydney, NSW Australia; 23 Beijing University of Posts and Telecommunications, Beijing, China

**Keywords:** Palaeontology, Biological anthropology, Genetics of the nervous system, Archaeology, Cognitive neuroscience

## Abstract

Fossil endocasts record features of brains from the past: size, shape, vasculature, and gyrification. These data, alongside experimental and comparative evidence, are needed to resolve questions about brain energetics, cognitive specializations, and developmental plasticity. Through the application of interdisciplinary techniques to the fossil record, paleoneurology has been leading major innovations. Neuroimaging is shedding light on fossil brain organization and behaviors. Inferences about the development and physiology of the brains of extinct species can be experimentally investigated through brain organoids and transgenic models based on ancient DNA. Phylogenetic comparative methods integrate data across species and associate genotypes to phenotypes, and brains to behaviors. Meanwhile, fossil and archeological discoveries continuously contribute new knowledge. Through cooperation, the scientific community can accelerate knowledge acquisition. Sharing digitized museum collections improves the availability of rare fossils and artifacts. Comparative neuroanatomical data are available through online databases, along with tools for their measurement and analysis. In the context of these advances, the paleoneurological record provides ample opportunity for future research. Biomedical and ecological sciences can benefit from paleoneurology’s approach to understanding the mind as well as its novel research pipelines that establish connections between neuroanatomy, genes and behavior.

## Abstract (web summary)

Recent advances in paleoneurology are collated together in this comprehensive review, linking neuroanatomy to genes and behavior. We provide guidance to the next generation of researchers to move the field forward.

## Introduction

What, if anything, can fossils tell us about the mind? Paleontologists draw links between anatomical details that are preserved in the fossil record (e.g., the dimensions of a bone) and functions (e.g., locomotion). We start from the premise that “minds are simply what brains do”^1^, but understanding minds through fossils has proven to be a tortuous task at the center of a very contentious topic. Brain tissue does not fossilize like teeth and bones do, so it is only possible to observe the internal cavity of the skull’s braincase – called the endocranium – to understand external brain anatomy of extinct species. Endocasts – naturally occurring or artificially manufactured internal casts of the endocranium that *look* a lot like brains – only provide information about a few gross anatomical features, whereas a comprehensive understanding of brain function needs to incorporate microstructure, gene expression, connectivity, biochemistry, physiology, and behavior, among others. Furthermore, the structural and functional bases of the cognitive abilities that differentiate humans from other species are not fully understood even in present day humans and primates.

The evolutionary history of the human brain is fundamental to understanding the modern human mind. As the field of brain evolution has drawn increasingly from biomedical methods such as neuroimaging and genomic analyses, it has also become clear that the field can contribute to human health and well-being. Aspects of brain structure and function that are particular to humans may be linked to our susceptibilities to neurodegeneration and mental health disorders. Evolutionary history forms a foundation for translating animal models of human conditions.

**Fig. 1:**
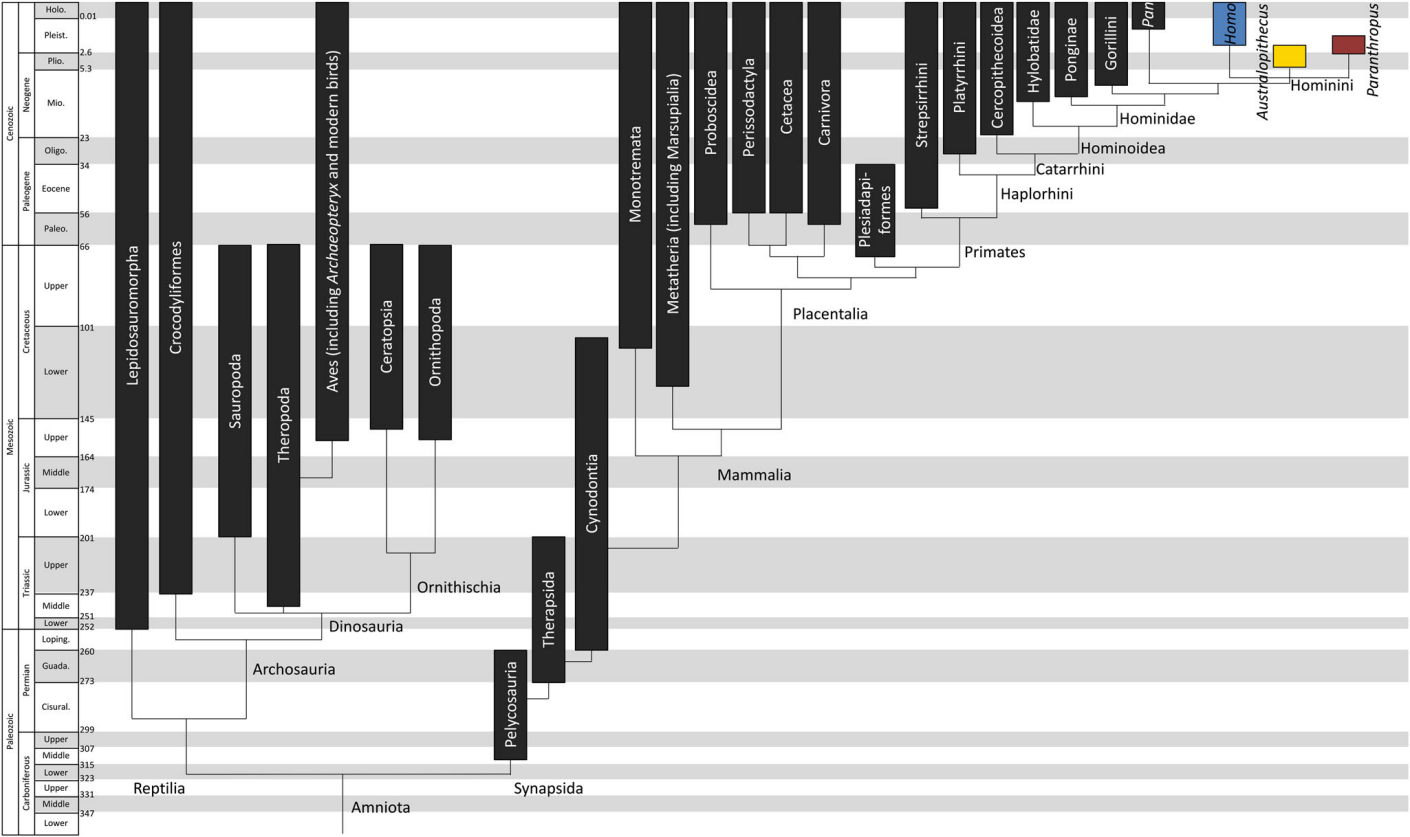
Time-scaled phylogenetic tree of amniotes. Included are taxonomic groups covering species discussed in this review and the accompanying “From Fossils to Mind” edited volume^304^. The geological time scale is from Cohen et al.^305^. Phylogeny and time ranges are based on Benton^306^, Kemp^307^, Sues^308^, and Kumar^309^.

**Fig. 2:**
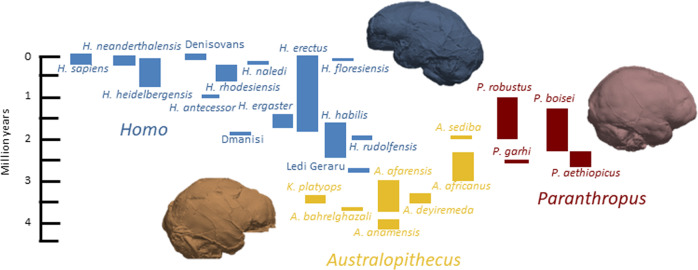
Hominin species time ranges and approximate taxonomic groupings into the three major hominin genera. In order of first appearance, *Australopithecus* (yellow), *Paranthropus* (red) and *Homo* (blue) species are plotted against a time scale, after Wood et al.^310^. Virtual endocasts are featured to represent each genus.

The evolutionary history of the species we discuss is represented in a phylogenetic tree (Fig. 1). The human brain is best understood through comparisons with closely related species of primates and other mammals, and also more distantly related species, which share both ancestral and convergent features with humans. The study of birdsong, for example, has provided essential insight into the neural mechanisms of vocal learning in birds, and its convergence with human language^2^. Early amniotes, including dinosaurs (early relatives of birds) and early synapsids (early relatives of mammals), provide a broader evolutionary perspective. Our own species (*Homo sapiens*) is a member of the great ape clade, also called hominids, along with three other great ape genera: *Pan* (*Pan troglodytes* - chimpanzees and *Pan paniscus* - bonobos), *Gorilla* (*Gorilla gorilla* - Western gorillas and *Gorilla beringei* - Eastern gorillas), and *Pongo* (*Pongo pygmaeus* - Bornean orangutans and *Pongo abelii* - Sumatran orangutans). The term “humans” refers to both fossil and present day *Homo sapiens*, and where relevant it can also include closely related archaic human species who have been shown to have made recent contributions to our genome. Hominids have among the largest brains of all animal species, except for the most massive ones – proboscideans (elephants) and cetaceans (whales and dolphins)^3^. Within hominids, our closest extant relatives are the chimpanzees and bonobos, with whom we shared our most recent common ancestor approximately 7–10 million years ago (Ma)^4,5^. Hominins include *Homo sapiens* and those fossil species that are more closely related to them than to chimpanzees and bonobos. There are numerous hominin species which evolved and became extinct over the past 6-7 million years, including species of the genus *Australopithecus*, from which the genus *Homo* likely arose approximately 2.8 Ma, as well as at least one other now extinct genus called *Paranthropus*^6^ (Fig. 2). In this review, we primarily focus on hominins, but also cover aspects of brain evolution in other species.

First, we review what we know about brain anatomy from the fossil record, and the state of the art in paleoneurology. Second, we discuss the gaps in our knowledge about brain evolution and highlight the need for more comparative research concerning energetics, function, and development. We seek to address big theoretical questions about what makes the modern human brain different not just from all other extant species, but even from the Neanderthals (*Homo neanderthalensis*), which are closely related archaic humans with whom *Homo sapiens* interbred for thousands of years^7,8^. These questions may have been asked for centuries, but answering them requires more comparative investigation of biological mechanisms. Third, we point to emerging directions in the field. We provide guidance to researchers to move the field forward. We indicate how mechanistic evidence from molecular and evolutionary biology can now be used to triangulate findings from studies of endocasts. We highlight the potential of new fossil and archeological discoveries and discuss how to best use neuroimaging methods to interpret anatomy from endocasts. Finally, we point to the possibilities that can arise from better data sharing and provide a link to an online list of tools and resources in neuroscience and paleontology.

## Data we can obtain from endocasts

The study of brain evolution in extinct animals based on their fossil remains was made possible by the introduction of endocasting techniques in paleoneurology^9–11^. The soft tissues of the nervous system rarely fossilize (with some exceptions^12,13^). As the brain grows and expands during ontogeny, its surrounding structures leave an imprint in the cranial bone. Endocranial imprints in fossil crania can therefore provide evidence about brain size, vasculature of the meninges, and aspects of brain morphology, including overall shape, sulcal patterns, and lateralization. It is notable that adult brains do not fill the entire cranial cavity, and that the space between the brain and the braincase is filled with cerebrospinal fluid, meninges, and blood vessels.

### Size

Brain size is mainly assessed indirectly through studies of endocranial volume, and thus depends on assumptions about the relationship of the brain to the braincase. Early on, greater brain size was assumed to represent higher intellectual capacities like memory or problem-solving abilities, and it was used to infer possible behaviors like sociality, complexity of the hierarchical system, the nutrient richness of the diet, and behavioral flexibility^14^. Brain size has long been a central consideration in understanding human evolution, especially because the human brain is large. The human brain is 3-4 times larger in volume than that of other hominids and appreciably larger than would be predicted by allometric scaling for a primate of similar body size^15^. Parallel to this, the archeological record provides evidence for increasing technological sophistication. However, the association between archeological artifacts and their makers is not always certain, and is constantly revised in light of new paleontological and archeological discoveries^16^. Members of the genus *Australopithecus* lived approximately 4.2–2 Ma and had adult endocranial volumes on average 20% larger than chimpanzees^17–19^. Historically, inclusion in the genus *Homo* required an arbitrary minimum brain size, as well as association with stone tools^20^, but this has since been updated due to evidence for toolmaking that predates the appearance of *Homo*^21^ (see discussion further on in the review). Also, two relatively recent species of *Homo*, *Homo floresiensis* and *Homo naledi*, had unexpectedly small endocranial volumes^22–24^. Trends in brain size increase have also been hypothesized in other mammalian lineages. According to Marsh^25^, during the Paleogene, all mammals had a small brain and there was a gradual increase in the size of the brain over evolutionary time, yet this was not the case in all clades. Likewise, reductions in brain size have been hypothesized in other vertebrate lineages. It is currently accepted that some species have smaller brains than their ancestors, including some primates^26^ and sauropod dinosaurs^27^. The ratio between brain size and body size is under dynamic developmental constraint and evolves independently, in terms of  direction and rate, in different vertebrate taxonomic groups^28^.

As absolute brain volume was shown not to be a reliable proxy of intelligence and/or cognitive ability across animals, Jerison^29^ proposed the use of an Encephalization Quotient (EQ) – the ratio between actual brain size and expected brain size (as derived from regression with body size among different reference species). The aim was to control for the allometric relationship between brain and body size, for relevant cross-species comparison of brain size variability. Comparisons between different clades have established that some groups display a mean EQ above 1, like carnivorans, cetaceans, primates, and birds. In dinosaurs, a modified measure of encephalization developed by Hurlburt^30^, the Reptile Encephalization Quotient (REQ), shows disparity between various clades. Sauropod and ceratopsian dinosaurs have values under 1, whereas ornithopod and theropod dinosaurs display higher values^31^.

Although relative brain size has been proposed to be an index of cognitive ability, the relationship between brain size and behavioral repertoire is hotly debated. Even after accounting for body size, some proboscideans and some cetaceans do have or have had brain sizes comparable to some representatives of the genus *Homo* (EQ of about 5), which should be considered when discussing the relevance of brain size to intelligence^32–34^. Furthermore, decades of research in primates have stressed a relationship between brain size and ecological factors^35,36^. However, social factors could be under explored due to greater difficulty in defining and obtaining relevant ethological data^37,38^, which could have more localized effects on brain organization^39,40^. These disparities can also be seen in examples from the vertebrate paleontological record. For example, the dwarf elephant of Sicily (*Palaeoloxodon falconeri*) reached an EQ of 5 and up to 7 on a purely herbivorous (low quality) diet^33^. Thus, the use of EQ as a proxy of intelligence is contentious, and other constructs have been proposed to better represent the mechanisms linking brain size and cognition^41^.

### Vasculature

**Fig. 3:**
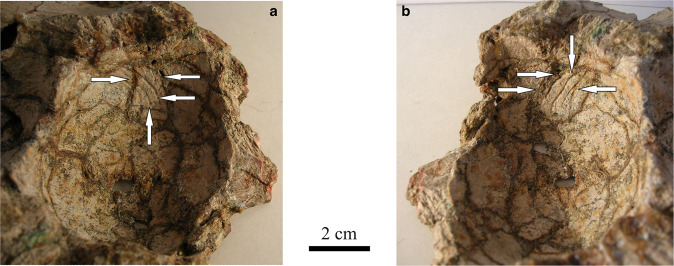
Valleculae on the frontal bone of a hadrosaurid dinosaur. **a, b** Internal surface of the frontal bone of a hadrosaurid dinosaur (*Amurosaurus riabinini*, AEHM 1/240) viewed on the left side and right side. Arrows point to some of the valleculae. (Lauters^311^, available at 10.5281/zenodo.7454914).

Cerebral blood flow is an indication of brain activity, including cognitive and other physiological processes. Blood flow to and within the brain in fossil specimens can be estimated from depressions made by blood vessels on the inner surface of the endocast, called valleculae, as well as from bony canals. Valleculae appear when the brain is in close contact with the bone surface, being more pronounced when the meninges are thin^9,42^. The observation of valleculae suggests a close apposition between brain surface and endocranial surface, indicating that the endocranium can be used to assess the size and shape of the brain in extinct vertebrate species. The space between the brain and skull is particularly noticeable in reptiles with smaller brains, including some extant lepidosauromorph reptiles such as sphenodons and iguanas. However, in most mammals and archosaurs (birds, dinosaurs, and alligators), the brain takes up a larger portion of the endocranial cavity and endocasts can serve as a reliable proxy for brain size and shape^43^. Although it had previously been assumed that dinosaur brains only occupy half of the endocranial cavity^29^, the discovery of valleculae has shown that some dinosaur brains were larger than previously estimated^30,44^. Among dinosaurs, Osmólska^45^ and Evans^46^, and later Godefroit et al.^47^ and Lauters et al.^48^, showed the presence of valleculae in Oviraptoridae, Pachycephalosauridae and Hadrosauridae (Fig. 3). Within hominins, a close correspondence between brain and endocranial sizes has long been assumed^29^. However, the degree to which the brain fills the cranial cavity varies among mammalian groups, with notable exceptions such as proboscideans^32^.

Vascular imprints in hominin endocasts and foramina through the cranial base have featured in discussions about taxonomy^49,50^, thermoregulation^51^ and blood flow rate^52^. Studies of the vascular imprints in fossil hominin endocasts identified specific patterns in early hominins, with *Paranthropus* being characterized by an enlarged occipital-marginal sinus, while the middle branch of the middle meningeal vessels cannot be found in *Australopithecus*^49,50^, but see^53^. The reorganization of the vascular system in early hominins has been interpreted by some as evidence for the emergence of a thermoregulation system for cooling down the brain under conditions of hyperthermia (the “radiator” theory^51^). Indeed, thermal stress might represent a severe biological and evolutionary constraint, especially if we consider the increase in brain size within the hominin lineage and the role of brain volume in heat production. Additionally, the size of the arteries, inferred from the size of the foramina in the cranial base, gives us an idea about blood flow and brain glucose utilization, a relationship which has been examined across mammals^52,54^. A study taking advantage of this correlation to infer brain metabolism reported a disproportionate increase of blood flow rate to the brain in fossil hominins^52^.

### Shape

All adult vertebrate brains have three major portions: a forebrain (prosencephalon), a midbrain (mesencephalon), and a hindbrain (rhombencephalon). In recent years, comparisons between vertebrate brains have benefitted from an updated nomenclature^55^ that reflects homology between structures (i.e., similarity due to evolutionary relationship). New methods, such as profiling neurons on the basis of transcriptomes, have provided new tests of homology^56^. However, differences in the relative size and shape of these portions and their components can make it difficult to directly compare the brains of distantly related species from fossils alone, since only the outer shape of the brain is preserved.

In most amniote species, such as dinosaurs, the forebrain, midbrain, and hindbrain are discernable on fossil endocasts. The cephalic and pontine flexures of the brain can be summarized as the angles between the forebrain and midbrain, and midbrain and hindbrain, respectively, and can be determined from endocranial surfaces. Such brain endocast flexure is known to vary substantially across species, but there is an overall trend toward greater flexure in small animals and less flexure in large animals^57^. This trend is also observed ontogenetically, where younger individuals have more marked flexures than mature individuals^58^. This is due to the negative allometric scaling of the brain, where smaller animals tend to have a proportionately larger brain that is more restricted by the size limits of the skull and spatial limits from other structures, such as the eyes. As a result, they tend to have increased flexures to accommodate the spatial restrictions of the skull^59^.

Discernable features of mammalian endocasts are disproportionately cerebral (i.e., corresponding to the pallium of the telencephalon). Due to the development of the neocortex in mammals, the outer surface is mainly telencephalon which covers structures of the diencephalon and midbrain, with the impressions of ventral hindbrain and cerebellum also visible in endocasts. With increased encephalization in primates, other brain parts are positioned mostly inferior to the cerebrum and contribute to a flexion at the cranial base.

In humans, the cerebrum is disproportionately even larger and more superiorly positioned, enveloping most of the brain except for the cerebellum (a structure of the hindbrain). The folding of the cerebrum into convolutions made up of sulci and gyri is partially represented on fossils. In addition, overall cerebral form can also be studied. The shape and size of the human cerebellum is also represented in endocasts^60^, however its very fine folds (called folia) are not imprinted on endocasts. A striking finding of cerebral shape comparison is that, while both present day and fossil *Homo sapiens* are similar in endocranial volume to Neanderthals, they have different endocranial shapes with only present day *Homo sapiens* being characterized by a “globular”^61^ endocranial shape. This globular shape is related to the emergence of taller frontal area, more bulging parietal areas, round occipital area housing a large and bulging cerebellum, and narrow and anterior-medially oriented temporal poles^62–64^. As part of this process, the basicranium became more flexed. The cranial base also flexes during the first year of life of extant humans^65^. This trait emerged relatively recently within the lineage^62,66,67^ and is addressed further on in the review.

### Cerebral sulci

The boundaries of cerebral cortical areas in fossil hominins are often assumed to be closely, if not exactly, associated with sulci which are sometimes evident in extant species^68–71^. Understanding the functional relevance of cerebral sulci and gyri is thus crucial for deriving as much information as possible from endocasts. It has been shown that the number of cortical areas increases with brain size across mammals^72–75^ as does the complexity of sulcal morphology across species^76,77^ and among humans^78^. The number of distinct cortical areas, however, does not appear to vary substantially with brain size among anthropoid primates. Homologues of human prefrontal cortex areas, for example, have been described in macaque monkeys^79^. In humans, the presence of the paracingulate sulcus as an additional fold in some subjects has been linked to subtle cytoarchitectural variation^80^ as well as behavioral^81^ differences (see Toro^77^ for a discussion). In macaques, thalamic inputs to the primary visual cortex have been found to be involved in the development of primary visual cortex histology as well as the positioning of the lunate sulcus^82^. Computer simulations have found that convolutions may occur at cortical area borders as a mechanical consequence of cortical growth^77^. This notion of mechanical stress is congruent with research relating the development of gyri to growth factors that increase neurogenesis^83^ as well as the finding that fold wavelength – the width of a fold – is conserved among primates^26^ given the relatively stable cortical thickness.

While we cannot test brain function in extinct species, we are able to use key features to make inferences about various functional brain regions and their boundaries. Two regions of the cortex differ considerably in sulcal morphology between humans and nonhuman primates and are of interest to paleoneurologists: the inferior frontal gyrus, in which Broca’s language area is situated, and the lunate sulcus, associated with changes in the size of the parietal association cortex relative to the occipital visual cortex^84,85^. When part of the cortex has expanded large enough to fold over and cover another one, the overhanging part is called an operculum. Developmental studies show humans have gained frontal and orbital opercula in the inferior frontal gyrus, making the morphology of the sulci more complex. Additionally, humans never develop a deep lunate sulcus covering other gyri as seen in nonhuman primates, making their sulcal boundaries more simplified in this region^86,87^.

It is a matter of debate in paleoanthropology to what extent evolutionary changes in sulcal patterns are causally linked to species differences in overall brain volume. Early australopiths had sulcal patterns more similar to extant great apes in form^19,53^, while later, larger-brained members of *Homo* had sulci that more closely resemble modern humans^17^, but the sulcal pattern in later australopiths and early *Homo* is still debated^88^. Clarifying the timing and functional consequences of these sulcal changes requires new resources, more fossils, more comparative data from extant ape brains for reference, and innovative approaches from other disciplines.

### Lateralization

Several brain functions in humans and many other vertebrates are lateralized^89^, which is reflected in associated lateralized behaviors^90^. Prime examples for asymmetrically organized brain functions are lateralized vocalization systems and limb preferences in many vertebrate species^91,92^. In many nonhuman primate species, for example, there is asymmetry in hand skill and preference related to the motor demands of different actions^93^. Behavioral lateralization in humans and nonhuman primates has been linked to hemispheric asymmetry in the anatomy of various brain regions, including the primary motor cortex and inferior frontal cortex^94,95^, as well as the morphology of the corpus callosum^96,97^. Indeed, it has been hypothesized that hemispheric specialization increased in hominins along with handedness, tool use and making, and language^90^.

While extant primates in general exhibit lateralization in these faculties, humans are thought to stand out by showing more consistent and more robust functional lateralization patterns^89^. The human cerebral cortex displays a larger degree and greater range of leftward directional asymmetry compared to chimpanzees, particularly in the posterior perisylvian region^98^. Given that lateralization of brain functions has been suggested to be associated with maintaining rapid conduction within intra-hemispheric circuits to support time-critical computational processes^99,100^, this difference could be considered an important step in human evolution as language and toolmaking capacities emerged^90^. However, it is a matter of debate which aspects of brain asymmetry are uniquely human, as very little is known about brain asymmetry in great apes beyond data from chimpanzees. To fill this gap, endocranial imprints captured from computed tomography (CT) scans have been studied as proxies for brains. For example, Neubauer et al.^101^ measured the magnitude and pattern of endocranial shape asymmetry in humans, gorillas, orangutans, and chimpanzees. They found that the magnitude of asymmetry was about the same in humans, gorillas, and orangutans. Chimpanzee endocasts, however, were less asymmetrical on average than those of their great ape and human relatives. These endocranial results corroborate Gómez-Robles et al.^98^ findings on the differences between chimpanzee and human brains as determined from magnetic resonance imaging (MRI) scans, but the broader comparative context suggests a different evolutionary interpretation: perhaps chimpanzees are unusually symmetrical among hominids. Neubauer et al.^101^ also examined the pattern of asymmetry and were able to show that not only humans, but also chimpanzees, gorillas, and orangutans exhibited the pattern of asymmetry previously described as typically human: the left occipital lobe, the right frontal lobe, and the right temporal pole and right cerebellar lobe protruded more relative to their contralateral parts. These results challenge the long-held notion that the human asymmetry pattern is unique.

Evidence of lateralization in fossils might improve our understanding of the emergence and development of complex cognitive abilities in hominins^102^. As summarized in Uomini and Ruck^103^, hand preferences can be determined from behavioral evidence such as cave paintings (e.g., proportions of left and right handprints found), stone tools (e.g., direction of traces of use on stone tools) and striations on fossil teeth (when they were used in gripping an object), from directional asymmetries measured on skeletons (e.g., arm bones), and from endocranial asymmetries. For example, evidence of right-handedness at the population-level was deduced from dental striation analyses in *Homo heidelbergensis*, *Homo neanderthalensis*, and fossil and present day *Homo sapiens*^104^. Furthermore, it was found that the average spatial asymmetry pattern on endocasts is shared among extant hominids and among fossil hominins^105^, but that humans show a pattern of asymmetry more variable than great apes, which may reflect increased functional and developmental modularization of the human brain^101^. Unfortunately, fossil evidence of behavioral and anatomical hemispheric asymmetries is scarce, and fossil preservation sometimes makes it difficult to observe and measure the underlying patterns of anatomical asymmetry.

## Unresolved questions

Theories about brain evolution center around these questions: What are big brains for? Could an increase in brain size serve different functions in different species? Could changes in the way brains function occur independently of changes in brain size? Could the development of large brains relate to plasticity, and in particular, culture? *We need to know what big brains do*. Humans claim the largest brain size (in weight and neuron number) relative to body size, with some aspects (e.g., surface area of association cortex) being particularly pronounced. To enrich our understanding of human brain size, it is important to consider how brain size is interpreted in distantly related species. We can gain insight into how large brains work by looking at convergent mechanisms of evolution in brain weight, neuron number, neuron density, energetics, and organization. Humans belong to one of only three groups of species that have brains over 700 g, along with whales and elephants. Birds also provide opportunities to study convergent mechanisms, and have much higher neuronal densities than mammals, with total neuron numbers that fall within primate range^106^. While the hominin fossil record provides the only direct evidence about encephalization in humans, a more thorough understanding of brain size changes could come from studying other vertebrates such as large-brained mammals, neuron-dense birds and their closest relatives (dinosaurs and crocodiles), as well as early synapsids, which allow us to draw deep comparisons between these groups.

The brain’s function as a “mind” depends on the whole body in which it is integrated. Sensory, motor, and physiological processes depend on the brain and the body together. Brain activity impacts the body beyond conducting mental functions, most obviously by consuming energy and producing heat. As such the paleoneurological evidence is best understood in the context of evidence from diverse research areas including comparative physiology and experimental archeology, among others.

### Energetics

Average endocranial volume increased during the evolution of the genus *Homo* from approximately 600 cm^3^ in *Homo habilis* to 1350 cm^3^ in present day *Homo sapiens*^62,107^. However, some later species, *Homo naledi* and *Homo floresiensis,* had less than half the brain mass of *Homo sapiens*. Besides the advantages of having a larger brain with more neurons providing more processing power, increase in brain size comes at a cost. Brain tissue is one of the most energetically demanding tissues in the mammalian body^108^. For example, in *Homo sapiens*, the adult brain requires 20% of the daily energy intake, even though it makes up only about 2% of the body mass^109^, and cerebral blood flow estimations based on the size of cranial foramina for some related mammalian species indicate that their brains are similarly expensive^54^. Remarkably, human children’s brains, at the time of peak formation of neuronal connections, consume more than 60% of the body’s resting metabolism^110^. Most of the energy funneled into the brain is claimed by its approximately 90 billion neurons, mostly for signaling processes and maintenance of resting potentials^111,112^. Notably, although roughly 80% of the human brain’s neurons are located in the cerebellum, this structure consumes only about 10% of the brain’s total energy. In contrast, the cerebral cortex contains roughly 20% of neurons and takes up nearly 60% of its energy^113,114^. This is due to the large, branched structure of many cortical neurons which have numerous synapses for integrating inputs and a large number of small unmyelinated axons with high conduction costs^115^. The number of synapses per neuron increases in association with brain size enlargement across primates, with humans predictably having cortical neurons with the greatest number of synapses^116^. Due to the lack of brain material from extinct hominin species, we can only speculate on neuronal numbers and associated energy costs. However, based on cranial foramen data from extant primates and fossil hominins, it has been estimated that blood flow increased steeply during the last 3 million years of human evolution, which is estimated to have seen a six-fold increase in total cerebral blood flow rate^52^. Additionally, neuron number in extant primates (including humans) scales almost linearly with brain mass, which would mean that the number of neurons might have more than doubled during evolution of the hominin lineage^111^.

How could humans afford such an increase in brain size and the corresponding increase in neuronal number while meeting the required energy budget? It was recently shown that avian species with similar or even higher neuron densities than primates^106,117^ show lower energy demands per neuron^118^. The modern human brain appears to achieve higher computational efficiency with a reduced energy budget due to reduction of voltage-gated potassium conductance in specific cortical neurons^119^. Direct measurements of whole-body metabolism indicate that *Homo sapiens* have a larger total energy expenditure and higher basic metabolic rates compared to other great apes, possibly indicating an overall increase of energy demands in hominin evolution^120^. This could be partly attributed to larger brains with more, and larger neurons. However, it should be noted that we are only beginning to understand how these species differ in brain cellular composition (including glia) and the molecular mechanisms that guide metabolic activity^121^. To meet the increasing energy demands of both body and brain, humans have adapted through both physiological and behavioral mechanisms to increase energetic turnover compared to great apes and this could potentially be related to brain size enlargement^120^. For example, data indicate an increase in body fat in modern humans combined with altered foraging strategies, more food sharing among individuals, and a less cost-intensive locomotion type^122^. The invention of fire, cooking, and other food preparation techniques might have made more calories available^123^, but see^124^. It has been suggested that these adaptations co-evolved with the increase of brain volume, and that they influence each other – for example, the higher processing capacities of larger brains allowed for more sophisticated foraging strategies and more cooperative social interactions which made more energy available to further promote brain growth. However, large and expensive brains could potentially pay for themselves in ways other than through increases in cognitive abilities. In fact, it has been suggested that large brains with increased numbers of glial cells might have evolved for thermoregulation in whales^125,126^. As discussed above, we have much yet to learn about how brain size relates to cognitive abilities.

How do brains operate on a different budget in humans compared to other species? It has been suggested that changes in metabolic rates reflect changes in cell composition and microstructure. To answer this question, we need more information about brain energetics in different species. We need to relate evidence from the archeological and paleoecological records of fossil hominids, such as how they extracted foods, to various changes in their brains. We can triangulate that information to further model aspects of cerebral metabolism for fossil species.

### Behavior and cognition

We do not have direct access to the behaviors of humans of the past, nor to the cognitive processes that underlie them, since these do not fossilize. It is, therefore, up to archeologists to infer their emergence and presence from the archeological record. The material culture of Paleolithic hominins includes tools^127–129^, graphic productions such as engravings and paintings^130–138^, objects of personal adornment^139–142^, non-utilitarian objects transported over long-distances^143,144^, and burials^145–148^, among others. Neuroscientific experiments conducted on modern participants have been used to infer the neural basis of the cognitive abilities needed by Paleolithic hominins to produce the artifacts that archeologists have discovered. Although these studies are valuable and informative, they necessarily involve present-day human subjects with present-day human brains performing replications of ancient behaviors. For example, it remains unclear which brain activity patterns in a *Homo erectus* individual corresponded to the production of hand axes. Pioneering studies have used functional neuroimaging to explore the neural basis of stone toolmaking and its potential co-evolution with language^149–152^. Other research has focused on the incremental working memory requirements for the successful knapping of stone tools from successive lithic industries^153–155^.

To date, we know very little about how our brains process potentially symbolic aspects of material culture such as ornaments, body paintings, and engravings which appeared in the archeological record long after the earliest stone tools. Functional MRI (fMRI) was recently used to investigate human brain activity while visually perceiving the earliest abstract engravings^156^. A follow-up study addressed the neural correlates of visually perceiving these abstract engravings when they have been attributed to a human maker^157^. Characterizing the cerebral bases of the cognitive processes implemented by modern participants to produce or perceive archeological material reveals information on the probable cognitive capacities and neural functions of earlier hominins. Nevertheless, this approach has some limitations. The strategies used by Paleolithic hominins and those used by modern participants may differ due to differences in environment and culture. To limit this bias, researchers have, for instance, controlled for the effect of using language to give task instructions^153^ or included a group of archeologists who are experts in Paleolithic engravings to mimic the familiarity fossil hominins had with this type of material^157^.

Paleolithic hominin brains depend on comparisons with other primate species as well. Unfortunately, there are few studies comparing functional brain organization across humans and other species using the same methods. Further, brain organization seems to differ between humans and other primates in regions relevant to understanding the neural bases of fossil hominin cognition. Thus far, it has been proposed that the early visual areas (V1, V2 and V3) show fewer differences between humans and rhesus macaques^158^ compared to intraparietal association areas that integrate multisensory information and function in the representation of tool use^159–161^. It has been suggested that species differences relate to developmental timing, and that more rostral areas, and in particular the prefrontal cortex which matures later ontogenetically, differ more between humans and other primates^162,163^. Understanding brain function in *Homo sapiens* and other extant species helps us to propose hypotheses on the evolution of hominin brain organization, and how it may relate to cognition and behavior.

**Fig. 4:**
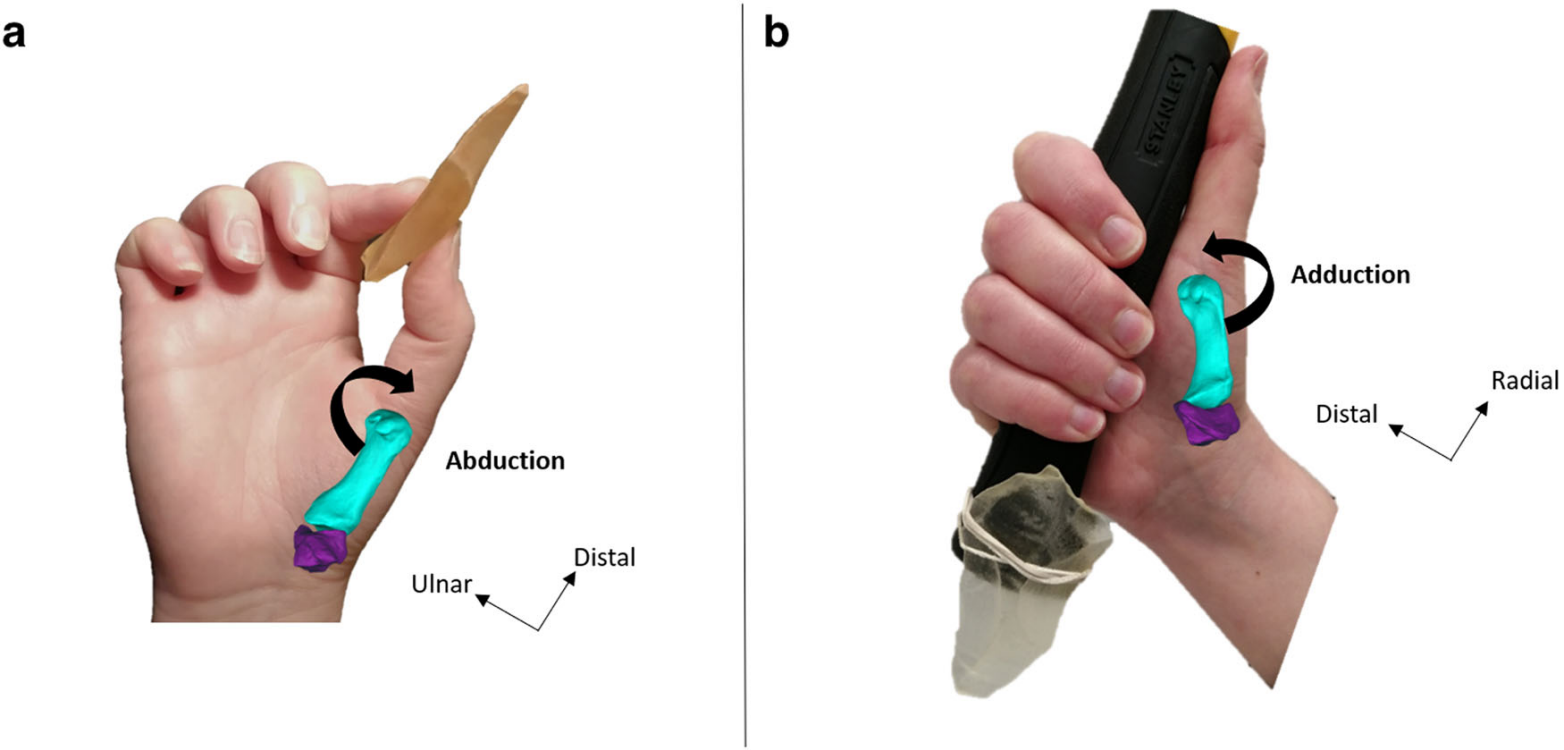
Examples of the relationship between hominin fossil hand morphology and early tool use. **a, b** A present day human’s hand demonstrating a precision grip when grasping an artifact (**a**) and a power “squeeze” grip when grasping a hafted artifact (**b**; both palmar views). Superimposed in turquoise (first metacarpal) and purple (trapezium) are the bones forming the trapeziometacarpal complex at the base of the thumb and responsible for its movements in a present day human (**a**) and a Neanderthal (Kebara 2; **b**). (Bardo^312^, available at 10.5281/zenodo.7452329).

Fossil hominins’ behavioral repertoires also depend on their postcranial anatomy. Fossil hand function is understood through the analysis of bone shape^164,165^ and musculoskeletal modeling^166,167^ and this is related to stone toolmaking ability through biomechanical studies^168,169^. For example, Neanderthal thumb morphology appears to be better suited for power “squeeze” grips than precision hand postures (although there is high interindividual variability^165^; Fig. 4). Experimental studies involving present-day human participants could further reveal how the brains and bodies of fossil hominins relate to the archeological record.

Are human brains specialized for toolmaking and other behaviors represented in the archeological record? How are their behavioral repertoires linked to brain organization, and are they independent of brain size? To answer this question, we need to know how brain structure relates to behavior in humans and other species. We can use that information to further model how fossil hominins could produce the artifacts they did, provided the brains they had.

### Development and plasticity

One of the aims of evolutionary developmental biology (known as Evo-Devo) is identifying developmental processes that support conservation and variation in structure and behavior. Much research in the field of human brain evolution has focused on identifying developmental processes specific to humans. One insight emerging from comparisons of ontogenetic trajectories across extant and extinct species is that the human brain grows *in utero* and during the first two years of postnatal life at a rapid rate for an extended period compared to chimpanzees. The extended duration in brain growth accounts for the relative enlargement of the adult human brain^170–173^. Another insight is that the human frontal cortex grows at a faster rate during the last trimester than it does in chimpanzees, and this cortical growth may be linked to differences in frontal lobe morphology^86,87,174–176^. While ontogenetic comparisons have yielded much insight into the evolution of the human brain, the paucity of fetal and postnatal brain samples available to study human and great apes has hindered progress in identifying developmental mechanisms accounting for structural and behavioral modifications in the human lineage.

Some new strategies have been developed to overcome the issue of small samples with the goal of providing a more complete characterization of the developmental mechanisms generating variation across human and non-human primates. The integration of information across scales of organization (e.g., behavior, anatomy, gene expression) is instrumental in finding corresponding ages across the lifespan of humans and chimpanzees, as well as other species^177^. This is because the sequence in behavior and biological maturation and aging are sufficiently similar across the two species that we can translate ages across their lifespan. Age alignments are possible throughout development to the end of life in chimpanzees. Importantly, most chimpanzees do not live past their forties, which equates to fifties to sixties in humans and subsequently there is a phase of life in humans (beyond fifties) with no clear counterpart in chimpanzees. Accordingly, chimpanzees should suffer from less cognitive decline towards the end of their lives than humans, and chimpanzee offspring may not benefit from the care of aged females as is evident in humans^178^. The evolution of lifespan has a range of consequences for species-specific cognition and social structures. As already mentioned, ontogenetic series of chimpanzee and human brains are difficult to obtain and hence limited, but a direct comparison of these series indicates that certain aspects of brain development, such as neocortical myelination, are protracted in humans compared to chimpanzees^179^. Other aspects of brain development, such as the timing of neocortical synaptogenesis, are shared by both species^180^.

Another way to overcome the very limited availability of ontogenetic series is by comparing patterns of variation observed in adult brains to infer certain aspects of development. For example, quantitative genetic analyses of large samples of chimpanzee and human adult brains indicate that human brain anatomy is more strongly influenced by environmental factors than chimpanzee brain anatomy, which is indicative of a higher level of developmental plasticity in humans^181,182^. While increased brain plasticity must have evolved in hominins, it is often considered to be associated with the evolution of secondary altriciality in humans^182–184^. Inferring levels of plasticity in fossil hominin species based on their patterns of anatomical variation is particularly challenging. There is, however, increasing evidence indicating that brain plasticity is influenced by genetic factors^185,186^ that can be identified and traced in hominin species through paleogenetic studies^187,188^. DNA methylation is an epigenetic mark that has been reconstructed in hominin ancient DNA (aDNA) samples which is highly correlated with gene expression and heritable through multiple cell divisions^189,190^. A better understanding of the molecular mechanisms in human-specific neuroplasticity could come from fossil hominins, since humans show substantial differential DNA methylation of genes involved in neurodevelopment and synaptic plasticity in comparison to great apes^191^.

Are human brains specialized for plasticity and an unusual propensity to be shaped by environmental variables? To answer this question, we need more information about brain development in different species. A better representation of neonatal fossil hominins could help us to better model how brain shape develops.

## Emerging directions

New directions are emerging for future research involving paleoneurological collections that incorporate neuroimaging, phylogenetic comparative methods, molecular biology, paleoanthropological discoveries, and sharing data and tools. These offer the potential to integrate information across different scales of organization from in vivo as well as in vitro systems to investigate biological mechanisms. New integrative frameworks allow for synergies and opportunities to answer unresolved questions about human brain evolution (Box 1).

Box 1 Summary of the kinds of data obtainable from the fossil record, unresolved questions about the origin of the human mind, and emerging directions that incorporate interdisciplinary research tools and innovative pipelines, making it possible to now answer those questions. Illustrating this is the natural partial endocast STS 60, from the collection at the Ditsong National Museum of Natural History, Pretoria, South Africa (photo on left) which preserves brain features that can then be compared to extant species (graphic on right; adapted from Heuer et al.^26^)

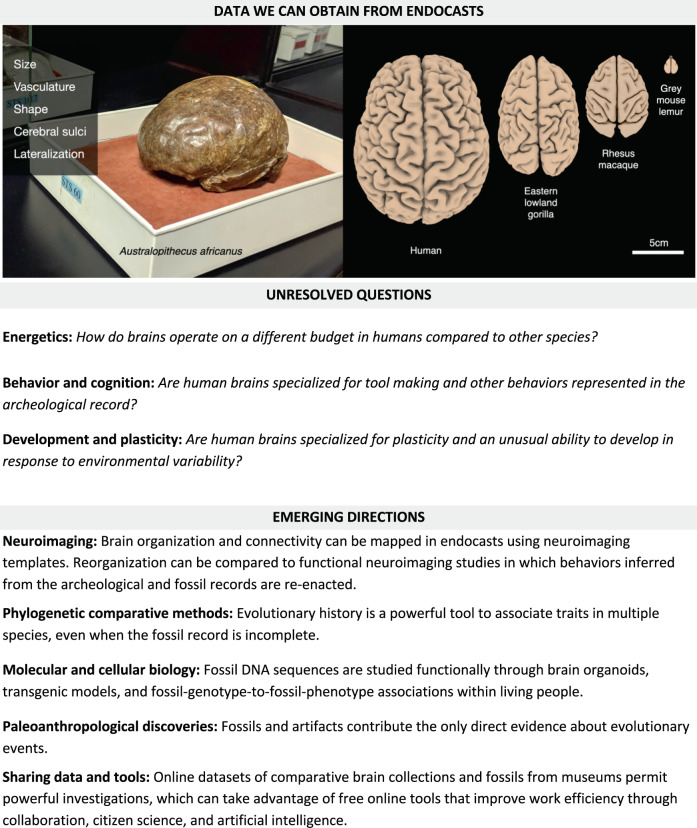

### Neuroimaging

Integrating endocast data with neuroimaging has opened an avenue to examine the evolution of the hominin brain in the context of the brain structure and function of present day humans. Neuroimaging techniques, such as MRI, are widely used to reconstruct and segment the human brain in three dimensions (3D). Combining data from neuroimaging and endocasts thus can be beneficial for examining the extent to which fossil endocast-derived features reflect aspects of brain structure. Early examples of such a combination include adapting metrics traditionally used on endocasts to MRI data^192,193^. For example, craniometric ratios of the LB1 (*Homo floresiensis*) endocast were compared to the normative distribution of the craniometric ratios of *Homo sapiens* derived from a large sample of MRI data, providing quantitative evidence of how LB1 deviates from *Homo sapiens*. In another illustrative example, Fournier et al.^194^ used brain MRI data from present day humans to reconstruct both the brain hull and the endocast, where consistent asymmetry patterns were observed. These findings suggest the feasibility of using endocasts to infer the evolution of brain asymmetry patterns.

Other studies further suggest that combining endocasts with neuroimaging makes it possible to investigate more comprehensive and localized morphometric characteristics of the brain, such as gyrification^195^. A recent study used endocasts and MRI data from the same sample of extant human subjects and revealed a close relationship between MRI-derived brain sulci patterns and the endocast-derived sulcal imprints in frontal, temporoparietal, and occipital regions^196^. These findings show a great potential to study the evolutionary process of structural reorganization in these cognitive-critical regions through comparisons of endocasts across species. A major aim of comparative studies is to infer the functional consequences of evolutionary changes in the hominin brain. In an exploratory study toward this goal, Kochiyama et al.^197^ reconstructed the Neanderthal ‘brain’ by deforming MRI-derived 3D human brain to the Neanderthal endocasts, to estimate the volume of distinct brain regions in both species. A prominent enlargement of the cerebellum was revealed in humans compared to Neanderthals (confirming earlier estimates using a different method^60^). They suggested that the enlarged cerebellum in *Homo sapiens* could relate to its function in cognitive abilities such as cognitive flexibility, attention, language processing, and episodic and working memory in present day humans. Cerebellar functions have been emphasized in light of recent findings supporting structural, size, and molecular changes in the cerebellum in humans^198–202^.

There are some caveats concerning the correspondence between endocasts and brains, such that information inferred from the endocast does not always parallel the organization of the brain. A recent study combining MRI and endocast data from humans and chimpanzees argued that evolutionary changes between the brains of humans and chimpanzees are disassociated with changes of neurocranial organization of the two species^203^. These findings seem to indicate that some aspects of brain organization are better reflected by endocranial anatomy than others. We thus advise verifying the examined characteristics using comparative endocast and neuroimaging data from primates before drawing a conclusion on brain evolution from the evidence of endocasts solely.

### Phylogenetic comparative methods

Phylogenies are hypotheses about the evolutionary relationships between species constructed from data such as genomes and fossil morphology (Fig 1). Species are more similar when they are closely related, a concept known as phylogenetic non-independence. Phylogenetic comparative methods comprise a statistical toolset that explicitly tackles this problem and, more generally, have the potential to improve inferences when different species are compared^204^. Although widely applied in evolutionary biology, phylogenetic comparative approaches have been used to a more limited extent in the study of fossil hominins^24^. Previous use of such approaches to understand brain evolution in fossil hominins concerns mainly brain size^205^ and encephalization^206^, but some aspects of variation in brain shape have been explored under this framework as well^207^. These approaches have the potential to help us infer ancestral states in the evolution of the hominin brain, as well as to reveal differences in evolutionary rates for different branches of the hominin phylogeny, which has been done for some cranial^208^ and dental traits^207^, and for some behavioral traits^209^. Fossil species can be included in ancestral state estimations even if their evolutionary relationships are not fully resolved, and make it possible to test models of evolution^210^. The inclusion of fossil data improves ancestral state estimations, as it calibrates values at nodes^211^. Hominin paleoneurology can also gain insight from studies of brain and body size scaling^212,213^ which use phylogenetic comparative methods to measure the convergence and divergence of traits across species, reconstruct ancestral states, and impute missing values. These approaches, however, are undeniably hampered by the uncertainty associated with the hominin phylogeny. Previous Bayesian analyses of the hominin phylogeny can be used to carry out these analyses^6,214^, but they are not free of controversy^215,216^. This phylogenetic framework, however, is likely to be improved and refined as more fossils are found and described in the future. In addition, the uncertainty associated with particular phylogenetic scenarios can be measured and included in calculations^6,214^.

Phylogenetic comparative approaches can be combined with other kinds of evidence to provide a more nuanced understanding of how traits are related. For example, quantitative methods of shape analysis (geometric morphometrics or other quantitative approaches) can provide a more nuanced description of endocranial shape variation over the hominin phylogeny^217^, in a similar way as it has been done to describe the evolution of endocranial shape in platyrrhine primates^218,219^. This approach, however, is still limited to the types of information that can be obtained from the endocranial evidence. Critical aspects of the microstructural, macrostructural and biochemical organization of the brain that are not preserved in endocasts cannot be reconstructed in fossil hominins through this strategy. Nonetheless, broader mammalian comparative contexts^220^, may still be useful to infer certain aspects of neurodevelopment in fossil hominins^221^, as well as the level of covariation between brain size and body size^222–224^, and other size- and shape-related variables^223^.

For example, hypothetical fossil brain structural connectivity could potentially be modeled from endocasts, even in the absence of any direct evidence. White matter tracts were reconstructed in the 100-year-old brain of the extinct Tasmanian tiger (*Thylacinus cynocephalus*) based on comparison with one other species, the Tasmanian devil (*Sarcophilus harrisii*)^225^. Structural brain connectomes have now been investigated in 125 mammalian species in comparison to phylogenetic distances^226^. Phylogenetic comparative methods could be used to improve predictions about brain structure in extinct hominins.

### Molecular and cellular biology

Inferences that have a direct genetic basis or that leave a record in individuals’ proteomes, may be testable in the future as more aDNA and paleoproteomic data become available. Indeed, aDNA analyses have revealed that *Homo sapiens*, Neanderthals and Denisovans shared coding changes in the *FOXP2* gene that can be related with certain forms of brain plasticity in these species^188,227,228^. Even when aDNA is not available, it is possible to ‘rebuild’ fossil genomes from the genomes of extant species using phylogenetic comparative methods, as has recently been done for the hypothetical mammalian common ancestor^229^. Such analyses present testable hypotheses about ancestors against which to compare future aDNA and paleoproteomic data derived from the fossil record.

New methodological advances based on the use of mouse models and brain organoids are now allowing researchers to test the functional significance of modern human-specific gene variants, including aspects of neurodevelopment^186,230^. Organoids, which are clusters of proliferating cells^231,232^, open new possibilities for comparative work across extant and extinct species, for which the primary brain tissue cannot be accessed. Organoids are generated from pluripotent stem cells (embryonic stem cells or induced pluripotent stem cells) and spun at high speed. These clusters of stem cells self-assemble into a collection of progenitor cells giving rise to various cell types^231^. Pluripotent stem cells can be induced from mature cells (e.g., skin cells) making organoids amenable for study in virtually any species. Crucially, the spatial patterns of gene expression in brain organoids are reminiscent of the brain’s spatial layout, thus facilitating species comparisons. Cells from organoids also form connections^233^. Based on these observations, it has been argued that organoids bear sufficient resemblance to nervous systems that they can be used to capture cross-species differences in developmental pathways.

Organoids have been instrumental in studying extinct species and extant species. Neanderthal alleles have been inserted into human pluripotent stem cell genomes to reveal how they impact brain development, opening up new ways to study genetic variation across extinct populations^234,235^. Organoids also permit studying developmental processes in extant nonhuman primates, including the most endangered species (e.g., orangutans, gorillas). Many, but not all, of recent studies have found that brain organoid maturation is slower in humans relative to extant great apes and monkeys^236–238^. The slower speed of human organoid maturation in vitro mirrors the extended duration of human brain growth relative to chimpanzees and other great apes observed in vivo. Yet, an unresolved question is how observations made from brain organoids translate to ground truth in primary tissue of the full organism. For example, it is unclear whether the media used to amplify pluripotent stem cells into organoids can impact observed species differences in gene expression, and rate of neuronal differentiation. We can now probe the maturation of the human brain at the single-cell level with single-cell RNA sequencing^239^. Accordingly, we know that brain organoids resemble but do not fully recapitulate the cell populations that are evident in human brain development. It is crucial to elucidate how well in vitro biological programs reflect in vivo biological programs^240^. Once those relationships are clarified, the integration of information from individuals, organoids, and scales of biological organization will provide exciting avenues to a better characterization of the mechanisms giving rise to characteristic features of the human brain.

A recent study used organoids to compare the role of the gene *Transketolase-like 1* (*TKTL1*) in corticogenesis of Neanderthals and *Homo sapiens*^241^. The findings point to differences in corticogenesis between Neanderthals and *Homo sapiens*, suggested to affect the frontal lobe, since there is high expression of the enzyme TKTL1 in the progenitors of the modern human frontal lobe. TKTL1 in *Homo sapiens* has a single amino-acid substitution (arginine) while the archaic form in Neanderthals, Denisovans, and other primates harbors a lysine^7^. TKTL1 in *Homo sapiens* specifically promotes the generation of basal radial glia progenitors that are key to primate cortical expansion via their increased capacity to generate neurons^242^. Since Neanderthals and *Homo sapiens* share similarly sized brains at adulthood, future research about development by molecular biologists, neuroscientists, and paleontologists could elucidate specific mechanisms that differ in their brain development.

New methods allow for inferences to be made about present-day human genetic variants derived from admixture with archaic humans. Such variants can be interpreted in the context of modern human brain and endocranial phenotypes. A globularity metric was established to describe the difference in endocranial globularity that has been observed based on the comparison of endocasts of *Homo sapiens* and Neanderthals^67^. The globularity metric was further examined using a large sample of MRI data from extant human subjects, suggesting a potential genotype-phenotype association of Neanderthal DNA alleles in the human genome to play a key role in variations in brain globularity. This approach to understand gene function could be extended to incorporate phenotypic data about brains, bodies, and behaviors, and potentially even take into account what we know about Neanderthal behavior from the archeological record.

### Paleoanthropological discoveries

As in all the sub-disciplines of paleontology, paleoneurology will benefit from the discovery of new specimens preserving either complete crania or natural endocasts. While they are relatively scarce, from time to time such specimens are unearthed and contribute to a better understanding of brain evolution. In parallel, cutting-edge methods, for example in virtual paleontology, provide exciting new data even from older finds.

Recent fossil discoveries have been used to investigate long-standing questions about the growth, organization, and shape of fossil hominin brains. Comparing the endocranial volumes of two *Australopithecus afarensis* infants dated to around 3.3 Ma to adult endocranial volumes of the same species, Gunz et al.^19^ suggested that these early hominins had a prolonged brain growth compared to chimpanzees after birth. Accordingly, the pattern of brain development that characterizes our species today may have emerged early within our lineage. Another breakthrough came from the discovery and comparative analysis of the endocasts of the earliest *Homo sapiens* including specimens from Morocco dated to 300,000 years^66,243^, which revealed a recent origin of the globular shape of the human brain^62^. Indeed, only fossils dated to less than 35,000 years fall within the range of shape variation observed in present-day humans^62,66,244^. Future discoveries of fossil crania representing the early ontogenetic stages of brain development will continue to shed light on when patterns of modern human brain development arose. Additional perinatal specimens will shed light on whether cranial shape features can be attributed to prenatal development versus childbirth, and on whether cranial shape of *Homo sapiens* is a result of facial gracilization^245,246^.

A recent study^88^ investigated a digitized sample of *Homo* endocasts from Africa and Eurasia using geometric morphometric methods to identify which brain regions had undergone differential expansions. The new analysis suggests reorganization of the inferior frontal gyrus (where Broca’s area is located) probably occurred after 1.8 Ma. Broca’s area is of particular interest due to its role in language, and it is also involved in tasks requiring complex hand movements such as toolmaking^247^. Such fossil findings could be further interpreted in the context of archeological evidence for the emergence of modern human behavior. The timing of this neuroanatomical change could be compared to changes in technology, hand anatomy, and other paleobiological factors. Using the archeological record as a guide, ancient behaviors that arose around 1.8 Ma could be replicated in laboratory studies that use physiological measures of brain and postcranial activity to determine the correspondence between Broca’s area and the behavior of fossil *Homo*.

The archeological record is a key marker of hominin brain and cognitive evolution. Recent archeological findings have indicated that the earliest use of stone tools was around 3.4 Ma^248^, and the intentional production of stone tools occurred by 3.3 Ma^21^. There is morphological evidence of human-like manipulation dated to around 3 Ma^249^.  All of these predate the appearance of the genus *Homo* by at least 200,000 years ago (*Homo sp*. remains from an unknown species have been dated to 2.8 Ma^250^). This implies that other hominins also had the ability to use and make stone tools.  This includes australopiths, whose hand morphology is close to that of non-human great apes^164,166^. The recent discovery of Oldowan tools associated with *Paranthropus* remains also supports the idea that non-*Homo* hominin species may have had the cognitive abilities for using and making tools^251^. Furthermore, archeological studies that applied recently available techniques, such as microwear analysis and spatial analysis, have contributed to  a new appreciation of the Neanderthals’ role in the production of bone tools and symbolic structures^252,253^. Future discoveries about fossil hominin material culture, functional anatomy, and neurobiology will continue to shed light on their technological and socio-cultural abilities.

### Sharing data and tools

**Fig. 5:**
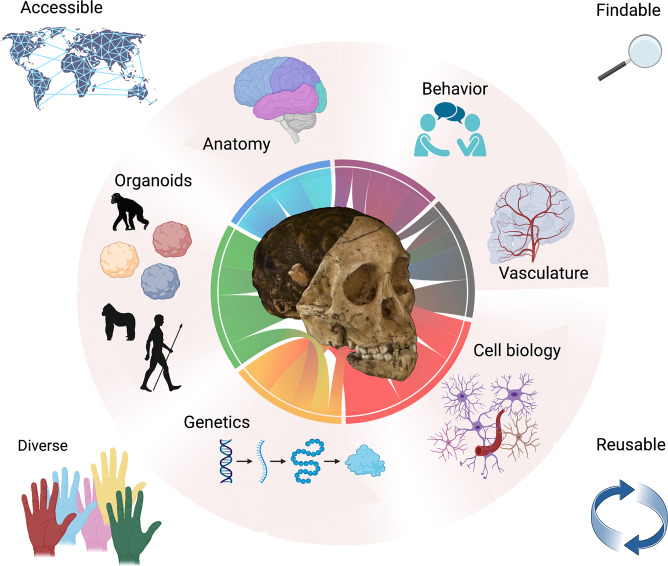
The “From Fossils to Mind” framework. Endocasts provide information about brain anatomy and vasculature in extinct hominin species, and evidence for their behavior can be gleaned from the archeological record. These data can be interpreted by integrating ancient DNA with new experimental methods at multiple scales (e.g., cell biology, anatomy, genetics, and organoids). To accelerate progress, researchers should collaborate in diverse teams and make their data accessible, findable, and reusable. Created with Biorender.com. Chord diagram done with Flourish (https://app.flourish.studio). The photo of the fossil cast in the center was composited from *Australopithecus africanus* - Cast of Taung child by Didier Descouens, available under CC BY-SA 4.0 at https://commons.wikimedia.org/wiki/File:Australopithecus_africanus_-_Cast_of_taung_child.jpg.

Investigations of complex phenomena, such as brain evolution, benefit from holistic, integrative approaches that synthesize diverse data from multiple sources, allowing researchers to address questions beyond the capabilities of any one group (Fig. 5). Research consortia are one solution to this challenge. ManyPrimates is an open grassroots consortium that collects comparative experimental psychological data about diverse primate species to address questions about the origin of the human mind using phylogenetic models^254^. In completing its first study, it has built a reproducible infrastructure for conducting broad-scale collaborative comparative research^255^. Despite this success, however, such big team science approaches face funding challenges and are not always free and openly available^256^.

More expansive approaches draw from open science principles^257^, reproducible research^258^ and FAIR data management practices^259^, alongside the CARE principles for indigenous data governance^260^. As we enter “the decade of digital brain”, the neuroscientific community is developing a concept to work with data to obtain new knowledge and model the brain^261^. The COVID-19 pandemic provided a case study on the scientific success achievable through collaborative as opposed to competitive knowledge generation emphasized by open science^262^. It follows that improvements in data integration accelerate progress through greater efficiencies, and more powerful analyses with greater dimensionality.

The transition to open science and integration represents a major shift in our epistemic culture (sensu Cetina^263^) and comprises two sets of challenges. First are the difficulties posed by social, political, and economic hurdles to open sharing. Across scientific fields, researchers are often reluctant to share data in order to maintain priority in publishing and commercial pursuits^264–267^. However, new generations are bringing fresh expectations regarding information sharing. Progress can be seen in revisions to the education of researchers, where preregistration, data management planning, and information science are increasingly incorporated into basic scientific training^268,269^. Similarly, journals, funding agencies, and academic societies are shifting their policies to encourage good practice in data sharing^270,271^. The challenges facing paleoneurology are further exacerbated by the fact that much of the primary fossil, archeological and comparative primatological data are distributed across institutions and cannot physically travel because of safety concerns, intellectual property issues, as well as international regulations pertaining to the export/import of biomaterials from endangered species and cultural artifacts. The COVID–19 pandemic has forced museums to rely more heavily on technology than they previously used to and has stressed the need to create and curate digital data as well as platforms that allow for remote accessibility. Creating digital replicas or proxies for the primary data, including microCT scans of fossils and artifacts, brain MRI scans and digitized histological sections, genetic sequences, and video captures of behavior, all make data more mobile and facilitate sharing. However, curating digital data in a museum comes with advantages as well as disadvantages^272^ that need to be addressed, especially if the process is to be carried on in a manner that most benefits local researchers and the custodians of the fossils in developing countries. Efforts at data sharing often run up against ethical issues emanating from the global power imbalances between developed and developing countries, imbalances that echo historic colonization and exploitation. The CARE principles provide a foundation for developing mutually beneficial systems of scientific exchange that respects the rights and authority of indigenous groups living and working with researchers in the field as well as local stakeholders such as the national museums, and cultural heritage authorities responsible for maintaining fossil and archeological collections.

The second major hurdle is technical. Once we can agree to share, how can we integrate the diverse types of data necessary for evolutionary studies of the mind, provided that the data originate from a multitude of sources, teams and domains of interest each with differing research praxes? Technical implementation of open science principles depends on a suite of interrelated best practices, standards and collaborative scientific infrastructure that provide open access to validated primary data. Data that are documented with standardized metadata and indexed can be easily located on the Web. The National Center for Biotechnology Information was established by the USA government to develop automated systems for storing molecular biology data and also provides tools to study them^273^. In another compelling example, the Global Biodiversity Information Framework, developed by the Organization for Economic Cooperation and Development^274,275^ aggregates primary species occurrence data based largely on databases maintained by the world’s museums and other research institutes. Each institution maintains its records independently, and shares them collaboratively using common data standards such as Darwin Core^276^. Similar systems are available for smaller academic research communities that can serve as model implementations for a collaborative paleoneurology community. These include efforts to systematize data collection protocols in primatology^277^. In paleoanthropology, Paleo Core maintains a data standard derived from Darwin Core, provides tools for field data collection, and an online platform for research teams to collaboratively manage their data^278–281^. Despite these examples, no overarching infrastructure exists for integrating the diverse neurobiological, molecular, anatomical, fossil, archeological, and behavioral data essential for studying the origin of the human mind. Establishing the necessary infrastructure will require better funding and resources^272,282,283^.

Recently, much progress has been made in making digital resources in neuroscience and paleontology freely available to the public. Here we highlight a selection of relevant resources that could be used in paleoneurology. Although we describe these and provide their URLs (Table 1), this is not an exhaustive list, and we also refer the reader to the curated and open Awesome Public Datasets, a repository that covers topics including Neuroscience, Museums, and Biology. All of the atlases, databases, tools, brain collections, resources, and software described here are listed in the webpages of the European Network for Brain Evolution Research, which also links to a mailing list on relevant topics.

**Table 1:** Directories, collections, tools, and database infrastructure described in the text. Arranged by material covered (italics).

Name	Website
DIRECTORIES / COMPILATIONS	
Awesome Public Datasets	https://github.com/awesomedata/awesome-public-datasets
European Network for Brain Evolution Research	https://sites.google.com/site/eurobrainevolnet
COLLECTIONS	
*Digitized museum collections*	
Royal Belgian Institute of Natural Sciences	https://virtualcollections.naturalsciences.be
*Digital endocasts and models*	
EndoMap	https://www.endomap.org
MorphoMuseum	https://morphomuseum.com
MorphoSource	https://www.morphosource.org
*Neuroimaging*	
Brain Analysis Library of Spatial maps and Atlases (BALSA)	https://balsa.wustl.edu
Brain Catalogue	https://braincatalogue.org
BrainBox: Brain Catalogue Primates	https://brainbox.pasteur.fr/project/BrainCataloguePrimates
Digital Brain Bank: Digital Brain Zoo	https://open.win.ox.ac.uk/DigitalBrainBank/#/datasets/zoo
National Chimpanzee Brain Resource	https://www.chimpanzeebrain.org
PRIMatE Data Exchange (PRIME-DE)	https://fcon_1000.projects.nitrc.org/indi/indiPRIME.html
*Digitized histology*	
BigBrain	https://bigbrainproject.org
BrainMaps.org	http://brainmaps.org
Comparative Mammalian Brain Collections	http://brainmuseum.org
*Brains (tissue / physical specimens)*	
National Chimpanzee Brain Resource	https://www.chimpanzeebrain.org
Primate Brain Bank	https://primatebrainbank.org
*Gene expression*	
Allen Brain Map	https://portal.brain-map.org
PsychEncode (comparative brain development)	http://www.evolution.psychencode.org
TOOLS	
*Endocast annotation*	
Curve-editor	https://gitlab.com/jeandumoncel/curve-editor
Endex	https://perso.liris.cnrs.fr/gilles.gesquiere/wiki/doku.php?id=endex
*Neuroimaging annotation*	
Brain Visa	https://brainvisa.info/web
BrainBox	https://brainbox.pasteur.fr
PRIMatE Resource Exchange (PRIME-RE)	https://prime-re.github.io
*Histological annotation*	
MicroDraw	https://microdraw.pasteur.fr
*Developmental age comparison*	
Translating Time	http://www.translatingtime.org
DATA INFRASTRUCTURE AND STANDARDS	
Darwin Core	https://dwc.tdwg.org
Global Biodiversity Information Facility (GBIF)	https://www.gbif.org
National Center for Biotechnology Information (NCBI)	https://www.ncbi.nlm.nih.gov
Paleo Core	https://paleocore.org

*Note*. This is not an exhaustive list. See directories / compilations above for more resources.

Sharing of digital endocast models is available through the digital repositories MorphoMuseum, with endocasts from extant and extinct species (e.g., humans, cercopithecoids, ungulates, cynodonts, dinosaurs, sloths, turtles), and MorphoSource (e.g., bats, rodents, primates, birds). Digitized museum collections, such as paleontological and anthropological 3D models that are available online from the Royal Belgian Institute of Natural Sciences, not only facilitate access to resources, but also allow for new morphometric *s*tatistical approaches to be more readily applied to endocasts. In line with this, the digital recognition of sulci using mathematical curvature and automated comparison is becoming more common^23,284^. Density maps that represent the probability of finding a specific sulcus in a human endocast^285^ can be viewed and downloaded from EndoMap. Endocasts can be automatically reconstructed from 3D models of dry crania using the software Endex^285^ and an algorithm for labeling detected sulci is freely available online^196^.

As endocasts become increasingly available in digital form, they benefit from comparison to the brains of extant species. An advantage of digital resources is that they can easily be combined in analyses, providing an opportunity for investigations to transcend disciplinary boundaries. Online brain collections allow for the comparison of structures in multiple species, and can be developed into algorithms to improve parcellations. Many of the brain collections are presented along with filters and search functions, and often include tools to annotate and measure them. Significant samples of chimpanzee, human, and other primate brain MRIs, covering the lifespan, have become available. For example, the Brain Analysis Library of Spatial maps and Atlases, an adjunct of the Human Connectome Project, includes MRI and fMRI data from humans and macaques. The National Chimpanzee Brain Resource includes a searchable directory of chimpanzee brain MRIs and physical specimens, with information about rearing, handedness, availability of cognitive test performance, and age. The Primate Data and Resource Exchange (Prime-DRE^286,287^) is a growing resource for structural, diffusion-weighted and functional MRI data from primates, mainly macaques, and tools to work with these data^288^. Many physical brain collections also feature online inventories and websites. The Primate Brain Bank provides brains for researchers. The Brain Catalogue allows users to interactively view MRI data and surface reconstructions, provides tools for collaborative segmentation, and gives open access to MRI data from a large number of vertebrate species. The Digital Brain Zoo allows users to preview and request structural MRI and diffusion MRI outputs for diverse mammalian species. The Allen Institute shares high-resolution histological and transcriptomic data from humans and mice as adults and in development. PsychENCODE provides human, macaque, and chimpanzee gene expression data in brain tissues over development.

Tools are publicly available to precisely map and measure brain structures from digital brain images. For example, BrainVisa is downloadable free software useful for functions such as mapping sulci. More recently, tools have been developed for online collaborative use. The Web app BrainBox enables researchers to collaborate in real-time on the segmentation of challenging datasets, such as developmental or cross species MRI data, to measure structures of interest and render 3D reconstructions on the fly in the browser^289^. The collection of structural MRI data from 34 primate species used to reconstruct surfaces in Heuer et al.^26^ is available on the BrainBox website together with their cerebral masks for easy access and community driven projects.

Histological collections are more demanding to create than MRIs, and require more storage space at high resolutions. Still, some histological collections are available online. Flatbed scans and photos of brains from the Comparative Mammalian Brain Collections spanning over 100 different species of mammals are available online. BrainMaps has comparative neurohistology for several species at microscopic resolution. MicroDraw enables collaborative annotation of digitized histological images. It is available online and supports large histological files, thus enabling researchers to work collaboratively on labor-intensive projects to annotate brain regions of interest on the basis of cellular organization. It has recently been used to provide a layer of collaboration to the Comparative Mammalian Brain Collection^290^. MicroDraw can also be used to work with BigBrain, the first openly accessible, microscopic resolution, 3D model of the human brain. Finally, algorithms utilizing multi-omic datasets are becoming available to facilitate comparison between different species brains and behaviors, with the potential to make life history predictions for fossil species as well. Translating Time is a free Web tool that incorporates the timing of abrupt transformations to translate ages across species. It can also be used to identify biological programs that vary in their timing in humans relative to most other species, including chimpanzees^177,220,291^.

Likewise, new computational techniques could address limitations in the study of endocasts. A limitation with understanding species from fossils alone is that their identifications and classifications are uncertain. Recently, artificial intelligence (AI) has been used to classify fossil specimens and develop taxonomies^292,293^. Also, labeling anatomical data is time consuming and subject to observer error. An efficient pipeline for processing large and complex anatomical image datasets currently used in the biomedical sciences is to crowdsource annotations using citizen science initiatives, and use these data to train deep learning models for human-level performance^294^. Automated taxonomic classifications and anatomical annotations could gain even more robust support from 3D geometric morphometry, and could be compared to vertebrate molecular evidence in the context of phylogeny. Reciprocally, paleoneurology can also inspire the development of AI itself by “reverse engineering” the minds of humans^295^, as well as other species, by documenting the changes that occurred during their natural histories.

We propose a set of guidelines to move the field forward (Box 2). Progress could be made for data sharing and availability by borrowing the advantages from one repository and extending them to others^296^. We need to develop more dynamic data repositories which allow users to input data themselves. There are challenges with species uncertainty in fossil and historical collections that could benefit from more extensive annotations. Another challenge to overcome is how to manage files of very large sizes, such as microscopic resolution histological images. Interdisciplinary dialogues and collaborations are essential to moving the field ahead.

Box 2 “From Fossils to Mind” recommendations for researchers Researchers interested in the origin of the human mind can do the following to accelerate discovery:

1. Contribute data and rich metadata in standardized formats to online databases that are open, FAIR, and CARE.
2. Where lacking, create new online databases, and provide a way for others in the community to continuously contribute relevant data.
3. Acquire long-term government and industry sponsorship for databases and large collaborations.
4. Develop comparative resources alongside applications (biomedical, and potentially ecological and cultural) to receive greater funding priority.
5. Collaborate in diverse teams to pool resources, data, and expertise about the species studied.
6. Collaborate inclusively with stakeholders, museums, local institutions, and local researchers in developed and developing countries.
7. Integrate open, FAIR, and CARE data sharing with university teaching and public outreach.

## Outlook

Paleoneurology has conjured up new ways to study the dead from within the living. Paleogeneticists, paleoanthropologists and archeologists have developed techniques to reconstruct long extinct species’ DNA, neuroanatomy, and behavioral repertoires using present day human participants. Novel methods have revealed associations between genotypes and phenotypes, and brains and behaviors, that can have a broader impact within the biomedical sciences (Fig. 5).

Although human brains share an overall similarity with the brains of other mammals, they remain an outlier in comparison to even our closest relatives in certain important parameters reviewed here. This has implications not only for human health, but also for understanding what is possible in terms of brain evolution. Regarding the former, human brain specializations make us vulnerable to brain disease. The increased size of our brain may lead to a predisposition to neurodegenerative disease^297^, the metabolic demands of our brains^298^ and the complex connectivity patterns^299^ may lead to a predisposition to schizophrenia, and our unique neurotransmitter^300^ and genetic profile^246,301^ leave us vulnerable to other mental health and substance use disorders^302^.

New techniques arising from the ecological, geological, and biomedical sciences provide paleontologists and archeologists with new opportunities, which in turn have the potential to inform broader applications. Fossil genomes can shed light on human disease. *Homo sapiens* – and the groups we recently admixed with – adapted to different environments around the world over time. Some of the alleles that are at present associated with human diseases such as auto-immune diseases, sickle cell disease, asthma, neuropsychiatric disorders, chronic kidney disease and obesity for example, might have previously been linked to these adaptations^301^. The study of Neanderthal-specific gene variants contained in modern human genomes in combination with endocast data from fossils provides the opportunity to glimpse into the effect that the gene variants have on phenotypic variation. Such observations from fossil endocast studies could in turn serve neuroimaging studies by defining new metrics of interest such as the globularity metric and determining new relationships between genotypes and phenotypes in modern humans. Brain organoids open a host of new possibilities for therapies and precision medicine, which can take advantage of the comparative work across extant and extinct species. Further progress can also be made by combining phenotypic and genotypic evidence in a phylogenetic framework, which adds statistical power to our understanding of how traits relate to each other beyond what is attainable from experimental studies on model systems alone^303^.

The brains of vertebrates have evolved on different trajectories in the diverse range of species that have lived on our planet. The fields of paleoneurology and evolutionary neurosciences have come a long way to uncover how the brains of each species of primate are different, how they are similar and how that relates to function and behavior in each respective environment. Progress is being made towards understanding how brain size, shape, macroanatomy, microanatomy, metabolism and development differ between extant species of hominids and uncovering the molecular neurobiology of extinct species.

Altogether, the combination of phylogenetic comparative approaches across the hominin phylogeny and in broader comparative contexts, the incorporation of new aDNA datasets, the use of new experimental approaches, the use of advanced methods to analyze the endocranium and determine its correspondence to neuroimages, new fossil discoveries, the interpretation of archeological evidence, and a more efficient approach to sharing the available resources offer new avenues to explore the evolution of the brain in fossil hominins (Fig. 5). This combination of approaches will allow us to move beyond long-standing debates on the significance of endocranial traits to a more informative scenario where a phylogenetically informed hypothesis will be testable using molecular and experimental data.

The deep perspective provided by bringing ‘fossils to mind’ allows for an intricate understanding of how neuroanatomy relates to genes and behavior, which in turn sets the context to study the same processes in the present, and even in the future (Box 3).

Box 3 Image representing the “From Fossils to Mind” workshop which led to the development of this paper

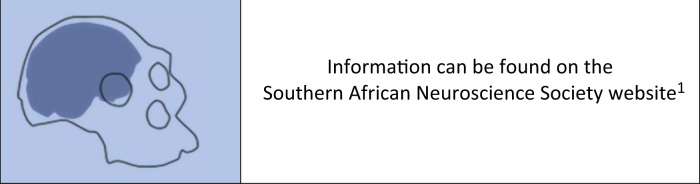

^1^ https://saneurosoc.co.za/fromfossilstomind

## References

[1] Minsky, M. *Society of Mind*. (Simon and Schuster, 1988).

[2] Pfenning AR (2014). Convergent transcriptional specializations in the brains of humans and song-learning birds. Science.

[3] Burger JR, George MA, Leadbetter C, Shaikh F (2019). The allometry of brain size in mammals. J. Mammal..

[4] Hallström BM, Janke A (2010). Mammalian evolution may not be strictly bifurcating. Mol. Biol. Evol..

[5] dos Reis M, Donoghue PC, Yang Z (2016). Bayesian molecular clock dating of species divergences in the genomics era. Nat. Rev. Genet..

[6] Dembo M, Matzke NJ, Mooers AO, Collard M (2015). Bayesian analysis of a morphological supermatrix sheds light on controversial fossil hominin relationships. Proc. Biol. Sci..

[7] Prufer K (2014). The complete genome sequence of a Neanderthal from the Altai Mountains. Nature.

[8] Kuhlwilm M (2016). Ancient gene flow from early modern humans into Eastern Neanderthals. Nature.

[9] Edinger T (1929). Die fossilen Gehirne. Ergebnisse der Anatomie und Entwicklungsgeschichte..

[10] Edinger T (1948). Paleoneurology versus comparative brain anatomy. Conf. Neurol..

[11] Kochetkova, V. I. *Paleoneurology*. (Wiley and Sons, 1978).

[12] Morton-Hayward AL (2020). A conscious rethink: Why is brain tissue commonly preserved in the archaeological record? Commentary on:. STAR: Science & Technol. Archaeol. Res..

[13] Figueroa, R. T. et al. Exceptional fossil preservation and evolution of the ray-finned fish brain. *Nature*, **614**, 486–491 (2023). 10.1038/s41586-022-05666-1 36725931

[14] Heldstab SA, Isler K, Graber SM, Schuppli C, van Schaik CP (2022). The economics of brain size evolution in vertebrates. Curr. Biol..

[15] Rilling JK (2006). Human and nonhuman primate brains: Are they allometrically scaled versions of the same design?. Evol. Anthropol..

[16] de Sousa A, Cunha E (2012). Hominins and the emergence of the modern human brain. Prog. Brain Res..

[17] Holloway, R. L., Broadfield, D. C., Yuan, M. S., Schwartz, J. H. & Tattersall, I. *The Human Fossil Record, Brain Endocasts: The Paleoneurological Evidence, Volume 3*. (2004).

[18] Kimbel, W., Rak, Y. & Johanson, D. C. *The skull of*Australopithecus afarensis. (Oxford University Press, 2004).

[19] Gunz P (2020). *Australopithecus afarensis* endocasts suggest ape-like brain organization and prolonged brain growth. Sci. Adv..

[20] Leakey LSB, Tobias PV, Napier JR (1964). A new species of the genus *Homo* from Olduvai Gorge. Nature.

[21] Harmand S (2015). 3.3-million-year-old stone tools from Lomekwi 3, West Turkana, Kenya. Nature.

[22] Falk D (2005). The brain of LB1, *Homo floresiensis*. Science.

[23] Holloway RL (2018). Endocast morphology of *Homo naledi* from the Dinaledi Chamber, South Africa. Proc. Natl. Acad. Sci ..

[24] Pestana, C., de Sousa, A. A., Todorov, O. S., Beaudet, A. & Benoit, J. Evolutionary history of hominin brain size and phylogenetic comparative methods. *Prog. Brain Res.* **275**, 217–232 (2023). 10.1016/bs.pbr.2022.12.014 36841569

[25] Marsh OC (1874). Small size of the brain in Tertiary mammals. J. Natural History..

[26] Heuer K (2019). Evolution of neocortical folding: A phylogenetic comparative analysis of MRI from 34 primate species. Cortex.

[27] Muller RT, Ferreira JD, Pretto FA, Bronzati M, Kerber L (2021). The endocranial anatomy of *Buriolestes schultzi* (Dinosauria: Saurischia) and the early evolution of brain tissues in sauropodomorph dinosaurs. J. Anat..

[28] Tsuboi M (2018). Breakdown of brain-body allometry and the encephalization of birds and mammals. Nat. Ecol. Evol..

[29] Jerison, H. J. *Evolution of the brain and intelligence*. (Academic Press, 1973).

[30] Hurlburt, G. R. *Relative brain size in recent and fossil amniotes: determination and interpretation*. (University of Toronto, 1996).

[31] Franzosa, J. W. *Evolution of the brain in Theropoda (Dinosauria)*. (The University of Texas at Austin, 2004).

[32] Benoit J (2015). A new method of estimating brain mass through cranial capacity in extinct proboscideans to account for the non-neural tissues surrounding their brain. J. Vertebrate Paleontol..

[33] Benoit J (2019). Brain evolution in Proboscidea (Mammalia, Afrotheria) across the Cenozoic. Sci Rep..

[34] Marino L, McShea DW, Uhen MD (2004). Origin and evolution of large brains in toothed whales. Anat. Rec A Discov. Mol. Cell Evol. Biol..

[35] Milton K (1981). Distribution patterns of tropical plant foods as an evolutionary stimulus to primate mental development. American Anthropol..

[36] DeCasien AR, Williams SA, Higham JP (2017). Primate brain size is predicted by diet but not sociality. Nat. Ecol. Evol..

[37] Lindenfors P, Wartel A, Lind J (2021). ‘Dunbar’s number’ deconstructed. Biol. Lett..

[38] Grabowski, M., Kopperud, B. T., Tsuboi, M. & Hansen, T. F. Both diet and sociality affect primate brain-size evolution. *Syst. Biol*. 10.1093/sysbio/syac075 (2022). 10.1093/sysbio/syac075 PMC10275546 36454664

[39] Todorov OS, Weisbecker V, Gilissen E, Zilles K, de Sousa AA (2019). Primate hippocampus size and organization are predicted by sociality but not diet. Proc. Biol. Sci..

[40] DeCasien AR, Barton RA, Higham JP (2022). Understanding the human brain: insights from comparative biology. Trends Cogn. Sci..

[41] van Schaik, C. P., Triki, Z., Bshary, R. & Heldstab, S. A. A farewell to the encephalization quotient: A new brain size measure for comparative primate cognition. *Brain Behav. Evol.* **96**, 1–12 (2021). 10.1159/000517013 34247154

[42] Hopson, J. A. in *Biology of the Reptilia* Vol. 9: Neurology A (eds C Gans, RG Northcutt, & P Ulinski) 39–146 (1979).

[43] Watanabe A (2019). Are endocasts good proxies for brain size and shape in archosaurs throughout ontogeny?. J. Anat..

[44] Hopson JA (1977). Relative Brain Size and Behavior in Archosaurian Reptiles. Annu. Rev. Ecol. Syst..

[45] Osmólska, H. Evidence on relation of brain to endocranial cavity in oviraptorid dinosaurs. *Acta Palaeontol. Polonica.* **49**, 321–324 (2004).

[46] Evans, D. C. New evidence on brain-endocranial cavity relationships in ornithischian dinosaurs. *Acta Palaeontol. Polonica.* **50**, 617–622 (2005).

[47] Godefroit, P., Escuillié, F., Bolotsky, Y. L. & Lauters, P. in *Bernissart dinosaurs and Early Cretaceous terrestrial ecosystems* 335-358 (Indiana University Press., 2012).

[48] Lauters P, Vercauteren M, Bolotsky YL, Godefroit P (2013). Cranial Endocast of the lambeosaurine hadrosaurid Amurosaurus riabinini from the Amur region, Russia. PLoS One..

[49] Saban R (1983). Les veines méningées moyennes des australopithèques. Bulletins et Mémoires de la Société d’Anthropologie de Paris..

[50] Falk D, Conroy GC (1983). The cranial venous sinus system in *Australopithecus afarensis*. Nature.

[51] Falk D (1990). Brain evolution in *Homo*: The “radiator” theory. Behav. Brain Sci..

[52] Seymour RS, Bosiocic V, Snelling EP (2016). Fossil skulls reveal that blood flow rate to the brain increased faster than brain volume during human evolution. R Soc. Open Sci..

[53] Beaudet A (2019). The endocast of StW 573 (“Little Foot”) and hominin brain evolution. J. Hum. Evol..

[54] Boyer, D. M. & Harrington, A. R. Scaling of bony canals for encephalic vessels in euarchontans: Implications for the role of the vertebral artery and brain metabolism. *J. Hum. Evol.* **114**, 85–101 (2018). 10.1016/j.jhevol.2017.09.003 29447763

[55] Reiner A (2004). Revised nomenclature for avian telencephalon and some related brainstem nuclei. J. Comp. Neurol..

[56] Hain D (2022). Molecular diversity and evolution of neuron types in the amniote brain. Science.

[57] Dudgeon TW, Maddin HC, Evans DC, Mallon JC (2020). The internal cranial anatomy of Champsosaurus (Choristodera: Champsosauridae): Implications for neurosensory function. Sci. Rep..

[58] Lautenschlager S, Hubner T (2013). Ontogenetic trajectories in the ornithischian endocranium. J. Evol. Biol..

[59] Platel, R. In Biology of the Reptilia Vol. 9: Neurology A (eds C. Gans, R. Northcutt, & P. Ulinski) 147-171 (1979).

[60] Weaver AH (2005). Reciprocal evolution of the cerebellum and neocortex in fossil humans. Proc. Natl. Acad. Sci..

[61] Lieberman DE, McBratney BM, Krovitz G (2002). The evolution and development of cranial form in *Homo sapiens*. Proc. Natl. Acad. Sci..

[62] Neubauer S, Hublin JJ, Gunz P (2018). The evolution of modern human brain shape. Sci. Adv..

[63] Bruner E (2004). Geometric morphometrics and paleoneurology: brain shape evolution in the genus. Homo. J. Hum. Evol..

[64] Bruner E, Manzi G, Arsuaga JL (2003). Encephalization and allometric trajectories in the genus *Homo*: evidence from the Neandertal and modern lineages. Proc. Natl. Acad. Sci..

[65] Neubauer S, Gunz P, Hublin JJ (2010). Endocranial shape changes during growth in chimpanzees and humans: a morphometric analysis of unique and shared aspects. J. Hum. Evol..

[66] Hublin JJ (2017). New fossils from Jebel Irhoud, Morocco and the pan-African origin of *Homo sapiens*. Nature.

[67] Gunz P (2019). Neandertal introgression sheds light on modern human endocranial globularity. Curr. Biol..

[68] Brodmann, K. *Vergleichende Lokalisationslehre der Grosshirnrinde in ihren Prinzipien dargestellt auf Grund des Zellenbaues*. (Barth, 1909).

[69] von Economo, C. F. & Koskinas, G. N. *Die cytoarchitektonik der hirnrinde des erwachsenen menschen*. (J. Springer, 1925).

[70] Bailey, P., Bonin, G. V. & McCulloch, W. S. *The isocortex of the chimpanzee*. (University of Illinois Press, 1950).

[71] Fischl B (2008). Cortical folding patterns and predicting cytoarchitecture. Cereb. Cortex..

[72] Bourgeois JP (1997). Synaptogenesis, heterochrony and epigenesis in the mammalian neocortex. Acta Paediatr..

[73] Changizi MA (2001). Principles underlying mammalian neocortical scaling. Biol. Cybern..

[74] O’Leary DD, Sahara S (2008). Genetic regulation of arealization of the neocortex. Curr. Opin. Neurobiol..

[75] Krubitzer L (2007). The magnificent compromise: cortical field evolution in mammals. Neuron.

[76] Prothero, J. W. & Sundsten, J. W. Folding of the cerebral cortex in mammals. A scaling model. *Brain Behav. Evol.* **24**, 152–167 (1984). 10.1159/000121313 6466964

[77] Toro, R. On the possible shapes of the brain. *Evol. Biol.* **39**, 600–612 (2012).

[78] Toro, R. et al. Brain size and folding of the human cerebral cortex. *Cereb. Cortex.* **18**, 2352–2357 (2008). 10.1093/cercor/bhm261 18267953

[79] Petrides M, Tomaiuolo F, Yeterian EH, Pandya DN (2012). The prefrontal cortex: comparative architectonic organization in the human and the macaque monkey brains. Cortex.

[80] Vogt BA, Nimchinsky EA, Vogt LJ, Hof PR (1995). Human cingulate cortex: surface features, flat maps, and cytoarchitecture. J. Comp. Neurol..

[81] Fornito, A. et al. Individual differences in anterior cingulate/paracingulate morphology are related to executive functions in healthy males. *Cereb. Cortex* **14**, 424–431 (2004). 10.1093/cercor/bhh004 15028646

[82] Dehay C, Giroud P, Berland M, Killackey H, Kennedy H (1996). Contribution of thalamic input to the specification of cytoarchitectonic cortical fields in the primate: effects of bilateral enucleation in the fetal monkey on the boundaries, dimensions, and gyrification of striate and extrastriate cortex. J. Comp. Neurol..

[83] Del-Valle-Anton L, Borrell V (2022). Folding brains: from development to disease modeling. Physiol Rev..

[84] Broca P (1861). Remarks on the seat of the faculty of articulated language, following an observation of aphemia (loss of speech). Bulletin de la Société Anatomique.

[85] Dart RA (1925). *Australopithecus africanus*: the Man-Ape of South Africa. Nature.

[86] Cunningham, D. J. *Contribution to the surface anatomy of the cerebral hemispheres*. (Academy House, 1892).

[87] Connolly, C. External Morphology of the Primate Brain: Springfield. *Ill.: Thomas* (1950).

[88] Ponce de Leon MS (2021). The primitive brain of early *Homo*. Science.

[89] Güntürkün O, Strockens F, Ocklenburg S (2020). Brain lateralization: A comparative perspective. Physiol. Rev..

[90] Rogers, L. J., Vallortigara, G. & Andrew, R. J. *Divided brains: the biology and behaviour of brain asymmetries*. (Cambridge University Press, 2013).

[91] Ocklenburg S, Strockens F, Gunturkun O (2013). Lateralisation of conspecific vocalisation in non-human vertebrates. Laterality.

[92] Strockens F, Gunturkun O, Ocklenburg S (2013). Limb preferences in non-human vertebrates. Laterality.

[93] Hopkins WD (2018). A review of performance asymmetries in hand skill in nonhuman primates with a special emphasis on chimpanzees. Prog. Brain Res..

[94] Becker Y (2022). Broca’s cerebral asymmetry reflects gestural communication’s lateralisation in monkeys (*Papio anubis*). eLife.

[95] Sha Z (2021). Handedness and its genetic influences are associated with structural asymmetries of the cerebral cortex in 31,864 individuals. Proc. Natl .Acad. Sci..

[96] Hopkins WD, Westerhausen R, Schapiro S, Sherwood CC (2022). Heritability in corpus callosum morphology and its association with tool use skill in chimpanzees (*Pan troglodytes*): Reproducibility in two genetically isolated populations. Genes Brain Behav..

[97] Westerhausen R, Papadatou-Pastou M (2022). Handedness and midsagittal corpus callosum morphology: a meta-analytic evaluation. Brain Struct. Funct..

[98] Gomez-Robles A, Hopkins WD, Sherwood CC (2013). Increased morphological asymmetry, evolvability and plasticity in human brain evolution. Proc. Biol .Sci..

[99] Ringo, J. L., Doty, R. W., Demeter, S. & Simard, P. Y. Time is of the essence: A conjecture that hemispheric specialization arises from interhemispheric conduction delay. *Cereb. Cortex.* **4**, 331–343 (1994). 10.1093/cercor/4.4.331 7950307

[100] Yang L (2022). Callosal fiber length scales with brain size according to functional lateralization, evolution, and development. J. Neurosci..

[101] Neubauer S, Gunz P, Scott NA, Hublin JJ, Mitteroecker P (2020). Evolution of brain lateralization: A shared hominid pattern of endocranial asymmetry is much more variable in humans than in great apes. Sci. Adv..

[102] Steele J, Uomini N (2009). Can the archaeology of manual specialization tell us anything about language evolution? A survey of the state of play. Cambridge Archaeol. J..

[103] Uomini NT, Ruck L (2018). Manual laterality and cognition through evolution: An archeological perspective. Prog. Brain Res..

[104] Lozano M (2017). Right-handed fossil humans. Evol. Anthropol..

[105] Balzeau A, Gilissen E, Grimaud-Herve D (2012). Shared pattern of endocranial shape asymmetries among great apes, anatomically modern humans, and fossil hominins. PLoS One..

[106] Strockens F (2022). High associative neuron numbers could drive cognitive performance in corvid species. J. Comp. Neurol..

[107] Robson SL, Wood B (2008). Hominin life history: reconstruction and evolution. J. Anat..

[108] Aiello LC, Wheeler P (1995). The expensive-tissue hypothesis: The brain and the digestive system in human and primate evolution.. Curr. Anthropol..

[109] Grande, F. Energy expenditure of organs and tissues. In *Assessment of energy metabolism in health and disease.* (ed. Kinney, J. M.) 88–92 (Ross Laboratories, 1980).

[110] Kuzawa CW (2014). Metabolic costs and evolutionary implications of human brain development. Proc. Natl. Acad. Sci..

[111] Herculano-Houzel S, Kaas JH (2011). Gorilla and orangutan brains conform to the primate cellular scaling rules: implications for human evolution. Brain Behav. Evol..

[112] Howarth C, Gleeson P, Attwell D (2012). Updated energy budgets for neural computation in the neocortex and cerebellum. J. Cereb. Blood Flow Metab..

[113] Kaufman, J. A. *Pattern and scaling of regional cerebral glucose metabolism in mammals*, Washington University in St. Louis, (2004).

[114] Azevedo FA (2009). Equal numbers of neuronal and nonneuronal cells make the human brain an isometrically scaled-up primate brain. J. Comp. Neurol..

[115] Wang SS (2008). Functional trade-offs in white matter axonal scaling. J. Neurosci..

[116] Sherwood CC (2020). Invariant synapse density and neuronal connectivity scaling in primate neocortical evolution. Cereb. Cortex..

[117] Olkowicz S (2016). Birds have primate-like numbers of neurons in the forebrain. Proc. Natl. Acad. Sci..

[118] von Eugen K (2022). Avian neurons consume three times less glucose than mammalian neurons. Curr. Biol..

[119] Beaulieu-Laroche L (2021). Allometric rules for mammalian cortical layer 5 neuron biophysics. Nature.

[120] Pontzer H (2016). Metabolic acceleration and the evolution of human brain size and life history. Nature.

[121] Jorstad, N. L. et al. Comparative transcriptomics reveals human-specific cortical features. *bioRxiv*, 2022.2009.2019.508480, 10.1101/2022.09.19.508480 (2022). 10.1126/science.ade9516 PMC10659116 37824638

[122] Navarrete A, van Schaik CP, Isler K (2011). Energetics and the evolution of human brain size. Nature.

[123] Gowlett JA (2016). The discovery of fire by humans: a long and convoluted process. Philos. Trans. R Soc. Lond B Biol. Sci..

[124] Cornelio AM, de Bittencourt-Navarrete RE, de Bittencourt Brum R, Queiroz CM, Costa MR (2016). Human brain expansion during evolution is independent of fire control and cooking. Front. Neurosci..

[125] Manger PR (2006). An examination of cetacean brain structure with a novel hypothesis correlating thermogenesis to the evolution of a big brain. Biol. Rev. Camb. Philos. Soc..

[126] Manger PR (2021). Amplification of potential thermogenetic mechanisms in cetacean brains compared to artiodactyl brains. Sci. Rep..

[127] Lepre CJ (2011). An earlier origin for the Acheulian. Nature.

[128] Lewis JE, Harmand S (2016). An earlier origin for stone tool making: implications for cognitive evolution and the transition to *Homo*. Philos. Trans. R Soc. Lond B Biol. Sci..

[129] Toth N, Schick K (2018). An overview of the cognitive implications of the Oldowan Industrial Complex. Azania: Archaeological Research in Africa..

[130] Aubert M (2014). Pleistocene cave art from Sulawesi, Indonesia. Nature.

[131] Henshilwood CS, d’Errico F, Watts I (2009). Engraved ochres from the Middle Stone Age levels at Blombos Cave, South Africa. J. Hum. Evol..

[132] Hoffmann DL (2018). U-Th dating of carbonate crusts reveals Neandertal origin of Iberian cave art. Science.

[133] Joordens JC (2015). *Homo erectus* at Trinil on Java used shells for tool production and engraving. Nature.

[134] Leder D (2021). A 51,000-year-old engraved bone reveals Neanderthals’ capacity for symbolic behaviour. Nat. Ecol. Evol..

[135] Li Z (2019). Engravings on bone from the archaic hominin site of Lingjing (Xuchang, Henan, China). Antiquity.

[136] Quiles A (2016). A high-precision chronological model for the decorated Upper Paleolithic cave of Chauvet-Pont d’Arc, Ardeche, France. Proc. Natl. Acad. Sci..

[137] Rodriguez-Vidal J (2014). A rock engraving made by Neanderthals in Gibraltar. Proc. Natl. Acad. Sci..

[138] Texier PJ (2010). From the Cover: A Howiesons Poort tradition of engraving ostrich eggshell containers dated to 60,000 years ago at Diepkloof Rock Shelter, South Africa. Proc. Natl. Acad. Sci..

[139] Caron F, d’Errico F, Del Moral P, Santos F, Zilhao J (2011). The reality of Neandertal symbolic behavior at the Grotte du Renne, Arcy-sur-Cure, France. PLoS One..

[140] d’Errico F, Henshilwood C, Vanhaeren M, van Niekerk K (2005). *Nassarius kraussianus* shell beads from Blombos Cave: evidence for symbolic behaviour in the Middle Stone Age. J. Hum. Evol..

[141] Steele TE, Álvarez-Fernández E, Hallet-Desguez E (2019). Personal ornaments in early prehistory a review of shells as personal ornamentation during the African Middle Stone. Age. PaleoAnthropol..

[142] Zilhao J (2010). Symbolic use of marine shells and mineral pigments by Iberian Neandertals. Proc. Natl. Acad. Sci..

[143] Benoit, J., Bednarik, R. & Helm, C. Manuports predate modern humans: A response to Wilkins et al. (2021). *S. Afr. Archaeol. Bull.* **77**, 76–78 (2022).

[144] Wilkins J (2021). Innovative *Homo sapiens* behaviours 105,000 years ago in a wetter Kalahari. Nature.

[145] Martinon-Torres M (2021). Earliest known human burial in Africa. Nature.

[146] Straus LG, Morales MRG, Cuenca-Solana D (2015). The Magdalenian human burial of El Miron Cave (Ramales de la Victoria, Cantabria, Spain): introduction, background, discovery and context. J. Archaeol. Sci..

[147] Vandermeersch, B. & Bar-Yosef, O. The Paleolithic burials at Qafzeh Cave, Israel. *Paléo* **30**, 256–275, (2019).

[148] d’Errico F, Backwell L (2016). Earliest evidence of personal ornaments associated with burial: the *Conus* shells from Border Cave. J. Hum. Evol..

[149] Stout D, Passingham R, Frith C, Apel J, Chaminade T (2011). Technology, expertise and social cognition in human evolution. Eur. J. Neurosci..

[150] Stout D, Toth N, Schick K, Chaminade T (2008). Neural correlates of Early Stone Age toolmaking: technology, language and cognition in human evolution. Philos. Trans. R Soc. Lond B Biol. Sci..

[151] Stout D, Chaminade T (2007). The evolutionary neuroscience of tool making. Neuropsychologia.

[152] Uomini NT, Meyer GF (2013). Shared brain lateralization patterns in language and Acheulean stone tool production: a functional transcranial Doppler ultrasound study. PLoS One..

[153] Putt SS, Wijeakumar S, Franciscus RG, Spencer JP (2017). The functional brain networks that underlie Early Stone Age tool manufacture. Nat. Hum. Behav..

[154] Putt SSJ, Wijeakumar S, Spencer JP (2019). Prefrontal cortex activation supports the emergence of early stone age toolmaking skill. Neuroimage.

[155] Stout D, Hecht E, Khreisheh N, Bradley B, Chaminade T (2015). Cognitive demands of lower paleolithic toolmaking. PLoS One..

[156] Mellet E (2019). Neuroimaging supports the representational nature of the earliest human engravings. R. Soc. Open Sci..

[157] Salagnon M, Cremona S, Joliot M, d’Errico F, Mellet E (2022). Neural correlates of perceiving and interpreting engraved prehistoric patterns as human production: Effect of archaeological expertise. PLoS One..

[158] Orban GA, Van Essen D, Vanduffel W (2004). Comparative mapping of higher visual areas in monkeys and humans. Trends Cogn. Sci..

[159] Bruner E, Battaglia-Mayer A, Caminiti R (2023). The parietal lobe evolution and the emergence of material culture in the human genus. Brain Struct. Funct..

[160] Peeters R (2009). The representation of tool use in humans and monkeys: common and uniquely human features. J. Neurosci..

[161] Mars RB (2011). Diffusion-weighted imaging tractography-based parcellation of the human parietal cortex and comparison with human and macaque resting-state functional connectivity. J. Neurosci..

[162] Teffer K, Semendeferi K (2012). Human prefrontal cortex: evolution, development, and pathology. Prog. Brain Res..

[163] Charvet CJ, Finlay BL (2014). Evo-devo and the primate isocortex: the central organizing role of intrinsic gradients of neurogenesis. Brain Behav. Evol..

[164] Dunmore CJ (2020). The position of *Australopithecus sediba* within fossil hominin hand use diversity. Nat. Ecol. Evol..

[165] Bardo A (2020). The implications of thumb movements for Neanderthal and modern human manipulation. Sci. Rep..

[166] Bardo A, Vigouroux L, Kivell TL, Pouydebat E (2018). The impact of hand proportions on tool grip abilities in humans, great apes and fossil hominins: A biomechanical analysis using musculoskeletal simulation. J. Hum. Evol..

[167] Domalain M, Bertin A, Daver G (2017). Was *Australopithecus afarensis* able to make the Lomekwian stone tools? Towards a realistic biomechanical simulation of hand force capability in fossil hominins and new insights on the role of the fifth digit. Comptes Rendus Palevol..

[168] Rolian C, Lieberman DE, Zermeno JP (2011). Hand biomechanics during simulated stone tool use. J. Hum. Evol..

[169] Macchi R (2021). Biomechanical demands of percussive techniques in the context of early stone toolmaking. J. R Soc. Interface..

[170] Leigh SR (2012). Brain Size Growth and Life History in Human Evolution. Evol. Biol..

[171] Sakai T (2012). Fetal brain development in chimpanzees versus humans. Curr. Biol..

[172] Cofran Z (2019). Brain size growth in. Australopithecus. J. Hum. Evol..

[173] Ponce de Leon MS, Bienvenu T, Akazawa T, Zollikofer CP (2016). Brain development is similar in Neanderthals and modern humans. Curr. Biol..

[174] Hurst SD (2022). Dmanisi, Malapa, and the evolution of Broca’s language area. Am. J. Biol. Anthropol..

[175] Mingazzini G (1928). Beitrag zur Morphologie der äusseren Grosshirnhemisphärenoberfläche bei den Anthropoiden (Schimpanse und Orang). Archiv. für Psychiatrie und Nervenkrankheiten..

[176] Walker AE, Fulton JF (1936). The external configuration of the cerebral hemispheres of the chimpanzee. J. Anat..

[177] Charvet CJ (2021). Cutting across structural and transcriptomic scales translates time across the lifespan in humans and chimpanzees. Proc. Biol. Sci..

[178] Hawkes K, Finlay BL (2018). Mammalian brain development and our grandmothering life history. Physiol. Behav..

[179] Miller DJ (2012). Prolonged myelination in human neocortical evolution. Proc. Natl. Acad. Sci..

[180] Bianchi S (2013). Synaptogenesis and development of pyramidal neuron dendritic morphology in the chimpanzee neocortex resembles humans. Proc. Natl. Acad. Sci..

[181] Gomez-Robles A, Hopkins WD, Schapiro SJ, Sherwood CC (2015). Relaxed genetic control of cortical organization in human brains compared with chimpanzees. Proc. Natl. Acad. Sci..

[182] Sherwood CC, Gómez-Robles A (2017). Brain Plasticity and Human Evolution. Annu. Rev. Anthropol..

[183] Hublin JJ, Neubauer S, Gunz P (2015). Brain ontogeny and life history in Pleistocene hominins. Philos. Trans. R Soc. Lond B Biol. Sci..

[184] Portmann, A. *A Zoologist Looks at Humankind*. (Columbia University Press, 1990).

[185] Charrier C (2012). Inhibition of SRGAP2 function by its human-specific paralogs induces neoteny during spine maturation. Cell.

[186] Enard W (2009). A humanized version of Foxp2 affects cortico-basal ganglia circuits in mice. Cell.

[187] Dennis MY (2012). Evolution of human-specific neural SRGAP2 genes by incomplete segmental duplication. Cell.

[188] Maricic T (2013). A recent evolutionary change affects a regulatory element in the human FOXP2 gene. Mol. Biol. Evol..

[189] Gokhman D (2014). Reconstructing the DNA methylation maps of the Neandertal and the Denisovan. Science.

[190] Mathov Y, Batyrev D, Meshorer E, Carmel L (2020). Harnessing epigenetics to study human evolution. Curr. Opin. Genet Dev..

[191] Guevara EE (2021). Comparative analysis reveals distinctive epigenetic features of the human cerebellum. PLoS Genet..

[192] Vannucci RC, Barron TF, Holloway RL (2011). Craniometric ratios of microcephaly and LB1, *Homo floresiensis*, using MRI and endocasts. Proc. Natl. Acad. Sci..

[193] de Sousa AA (2010). Hominoid visual brain structure volumes and the position of the lunate sulcus. J. Hum. Evol..

[194] Fournier, M., Combès, B., Roberts, N., Braga, J. & Prima, S. In *Medical Imaging 2011: Image Processing*. 309-315 (SPIE).

[195] Kobayashi, Y. et al. In *Dynamics of Learning in Neanderthals and Modern Humans Volume 2* 131-137 (Springer, 2014).

[196] Dumoncel J (2021). Are endocasts reliable proxies for brains? A 3D quantitative comparison of the extant human brain and endocast. J. Anat..

[197] Kochiyama T (2018). Reconstructing the Neanderthal brain using computational anatomy. Sci. Rep..

[198] MacLeod CE, Zilles K, Schleicher A, Rilling JK, Gibson KR (2003). Expansion of the neocerebellum in Hominoidea. J. Hum. Evol..

[199] Harrison PW, Montgomery SH (2017). Genetics of Cerebellar and Neocortical Expansion in Anthropoid Primates: A Comparative Approach. Brain Behav. Evol..

[200] Magielse, N., Heuer, K., Toro, R., Schutter, D. & Valk, S. L. A comparative perspective on the cerebello-cerebral system and its link to cognition. *Cerebellum*, 10.1007/s12311-022-01495-0 (2022). 10.1007/s12311-022-01495-0 PMC10657313 36417091

[201] Magielse, N. et al. Primate Cerebellar Scaling in Connection to the Cerebrum: A 34-Species Phylogenetic Comparative Analysis. *bioRxiv*, 2023.2003.2015.532597, 10.1101/2023.03.15.532597 (2023).

[202] Sereno MI (2020). The human cerebellum has almost 80% of the surface area of the neocortex. Proc. Natl. Acad. Sci..

[203] Alatorre Warren JL, Ponce de Leon MS, Hopkins WD, Zollikofer CPE (2019). Evidence for independent brain and neurocranial reorganization during hominin evolution. Proc. Natl. Acad. Sci..

[204] Nunn, C. L. *The comparative approach in evolutionary anthropology and biology*. (University of Chicago Press, 2011).

[205] Pagel, M. in *Morphology, shape and phylogeny* Vol. 269 (eds Norman MacLeod & Peter L. Forey) 286 (CRC Press, 2002).

[206] Puschel HP, Bertrand OC, O’Reilly JE, Bobe R, Puschel TA (2021). Divergence-time estimates for hominins provide insight into encephalization and body mass trends in human evolution. Nat. Ecol. Evol..

[207] Gomez-Robles A, Smaers JB, Holloway RL, Polly PD, Wood BA (2017). Brain enlargement and dental reduction were not linked in hominin evolution. Proc. Natl. Acad. Sci..

[208] Schroeder L, Roseman CC, Cheverud JM, Ackermann RR (2014). Characterizing the evolutionary path(s) to early *Homo*. PLoS One..

[209] Organ C, Nunn CL, Machanda Z, Wrangham RW (2011). Phylogenetic rate shifts in feeding time during the evolution of *Homo*. Proc. Natl. Acad. Sci..

[210] Slater GJ, Harmon LJ, Alfaro ME (2012). Integrating fossils with molecular phylogenies improves inference of trait. Evolution.

[211] Mongiardino Koch N, Garwood RJ, Parry LA (2021). Fossils improve phylogenetic analyses of morphological characters. Proc. Biol. Sci..

[212] Todorov OS (2021). Testing hypotheses of marsupial brain size variation using phylogenetic multiple imputations and a Bayesian comparative framework. Proc. Biol. Sci..

[213] Smaers JB (2021). The evolution of mammalian brain size. Sci. Adv..

[214] Dembo M (2016). The evolutionary relationships and age of *Homo naledi*: An assessment using dated Bayesian phylogenetic methods. J. Hum. Evol..

[215] Mongle CS, Pugh KD, Strait DS, Grine FE (2022). Modelling hominin evolution requires accurate hominin data. Nat. Ecol. Evol..

[216] Puschel HP, Bertrand OC, Reilly JEO, Bobe R, Puschel TA (2022). Reply to: Modelling hominin evolution requires accurate hominin data. Nat. Ecol. Evol..

[217] Sansalone G (2023). *Homo sapiens* and Neanderthals share high cerebral cortex integration into adulthood. Nat. Ecol. Evol..

[218] Aristide L (2015). Encephalization and diversification of the cranial base in platyrrhine primates. J. Hum. Evol..

[219] Aristide L (2016). Brain shape convergence in the adaptive radiation of New World monkeys. Proc. Natl. Acad. Sci..

[220] Workman AD, Charvet CJ, Clancy B, Darlington RB, Finlay BL (2013). Modeling transformations of neurodevelopmental sequences across mammalian species. J. Neurosci..

[221] Gomez-Robles A, Smaers JB, Sherwood CC (2017). The evolution of human altriciality and brain plasticity in comparative context. Am. J. Phys. Anthropol..

[222] Grabowski M (2016). Bigger brains led to bigger bodies?: The correlated evolution of human brain and body size. Curr. Anthropol..

[223] Miller IF, Barton RA, Nunn CL (2019). Quantitative uniqueness of human brain evolution revealed through phylogenetic comparative analysis. eLife.

[224] Montgomery SH, Capellini I, Barton RA, Mundy NI (2010). Reconstructing the ups and downs of primate brain evolution: implications for adaptive hypotheses and *Homo floresiensis*. BMC Biol..

[225] Berns GS, Ashwell KW (2017). Reconstruction of the cortical maps of the Tasmanian tiger and comparison to the Tasmanian devil. PLoS One.

[226] Faskowitz J (2022). Connectome topology of mammalian brains and its relationship to taxonomy and phylogeny. Front. Neurosci..

[227] Bornschein, U., Zeberg, H., Enard, W., Hevers, W. & Pääbo, S. Functional dissection of two amino acid substitutions unique to the human FOXP2 protein. *Sci. Rep.* **13**, 3747 (2023). 10.1038/s41598-023-30663-3 PMC9988825 36879029

[228] Atkinson, E. G. et al. No evidence for recent selection at FOXP2 among diverse human populations. *Cell* **174**, 1424-1435.e1415 (2018). 10.1016/j.cell.2018.06.048 PMC6128738 30078708

[229] Damas J (2022). Evolution of the ancestral mammalian karyotype and syntenic regions. Proc. Natl. Acad. Sci..

[230] Mora-Bermudez F (2022). Longer metaphase and fewer chromosome segregation errors in modern human than Neanderthal brain development. Sci. Adv..

[231] Fernandes S, Klein D, Marchetto MC (2021). Unraveling Human Brain Development and Evolution Using Organoid Models. Front. Cell Dev. Biol..

[232] Velasco S, Paulsen B, Arlotta P (2020). 3D brain organoids: Studying brain development and disease outside the embryo. Annu. Rev. Neurosci..

[233] Camp JG (2015). Human cerebral organoids recapitulate gene expression programs of fetal neocortex development. Proc. Natl. Acad. Sci..

[234] Kaluthantrige Don F, Kalebic N (2022). Forebrain organoids to model the cell biology of basal radial glia in neurodevelopmental disorders and brain evolution.. Front. Cell Dev. Biol..

[235] Trujillo CA (2021). Reintroduction of the archaic variant of NOVA1 in cortical organoids alters neurodevelopment. Science.

[236] Pollen AA (2019). Establishing cerebral organoids as models of human-specific brain evolution. Cell.

[237] Schörnig, M. et al. Comparison of induced neurons reveals slower structural and functional maturation in humans than in apes. *eLife* **10**, e59323 (2021). 10.7554/eLife.59323 PMC7870144 33470930

[238] Kanton S (2019). Organoid single-cell genomic atlas uncovers human-specific features of brain development. Nature.

[239] Bhaduri A (2021). An atlas of cortical arealization identifies dynamic molecular signatures. Nature.

[240] Andrews MG, Kriegstein AR (2022). Challenges of organoid research. Annu. Rev. Neurosci..

[241] Pinson A (2022). Human TKTL1 implies greater neurogenesis in frontal neocortex of modern humans than Neanderthals. Science.

[242] Taverna E, Gotz M, Huttner WB (2014). The cell biology of neurogenesis: toward an understanding of the development and evolution of the neocortex. Annu. Rev. Cell Dev. Biol..

[243] Richter D (2017). The age of the hominin fossils from Jebel Irhoud, Morocco, and the origins of the Middle Stone Age. Nature.

[244] Weber GW (2020). Before the massive modern human dispersal into Eurasia: A 55,000-year-old partial cranium from Manot Cave, Israel. Quaternary International..

[245] Zollikofer CPE (2022). Endocranial ontogeny and evolution in early *Homo sapiens*: The evidence from Herto, Ethiopia. Proc. Natl. Acad. Sci..

[246] Ganapathee DS, Gunz P (2023). Insights into brain evolution through the genotype-phenotype connection. Prog. Brain Res..

[247] Stout D, Chaminade T (2012). Stone tools, language and the brain in human evolution. Philos. Trans. R Soc Lond B Biol. Sci..

[248] McPherron SP (2010). Evidence for stone-tool-assisted consumption of animal tissues before 3.39 million years ago at Dikika, Ethiopia. Nature.

[249] Skinner MM (2015). Human evolution. Human-like hand use in *Australopithecus africanus*. Science.

[250] Villmoare B (2015). Paleoanthropology. Early *Homo* at 2.8 Ma from Ledi-Geraru, Afar, Ethiopia. Science.

[251] Plummer TW (2023). Expanded geographic distribution and dietary strategies of the earliest Oldowan hominins and *Paranthropus*. Science.

[252] Jaubert J (2016). Early Neanderthal constructions deep in Bruniquel Cave in southwestern France. Nature.

[253] Soressi M (2013). Neandertals made the first specialized bone tools in Europe. Proc. Natl. Acad. Sci..

[254] Many Primates et al. Establishing an infrastructure for collaboration in primate cognition research. *PLoS One.* **14**, e0223675 (2019). 10.1371/journal.pone.0223675 PMC6812783 31648222

[255] Many Primates et al. The evolution of primate short-term memory. *Anim. Behav. Cogn.* **9**, 428–516 (2022).

[256] Coles NA, Hamlin JK, Sullivan LL, Parker TH, Altschul D (2022). Build up big-team science. Nature.

[257] Allen, P. 2011. "Why we chose 'Open Science'". The Wall Street Journal, 30 November.

[258] Peng RD (2011). Reproducible research in computational science. Science.

[259] Wilkinson MD (2016). The FAIR Guiding Principles for scientific data management and stewardship. Sci. Data..

[260] Carroll, S. R. et al. The CARE principles for indigenous data governance. *Data Sci. J.* **19**, 1–12 (2020).

[261] Amunts, K. et al. *The coming decade of digital brain research - A vision for neuroscience at the intersection of technology and computing*, 10.5281/zenodo.6345820 (2023). 10.1162/imag_a_00137 PMC12247565 40800542

[262] Ellemers N (2021). Science as collaborative knowledge generation. Br. J. Soc. Psychol..

[263] Cetina, K. K. *Epistemic Cultures: How the Sciences Make Knowledge*. (Harvard University Press, 1999).

[264] Mulligan CJ, Boyer DM, Turner TR, Delson E, Leonard WR (2022). Data sharing in biological anthropology. Am. J. Biol. Anthropol..

[265] Hutson M (2022). Taking the pain out of data sharing. Nature.

[266] Tenopir C (2011). Data sharing by scientists: practices and perceptions. PLoS One..

[267] Nosek BA (2015). SCIENTIFIC STANDARDS. Promoting an open research culture. Science.

[268] Rhodes, E. In *The Psychologist* Vol. 30 14-15 (The British Psychological Society, 2017).

[269] Azevedo F (2022). Towards a culture of open scholarship: the role of pedagogical communities. BMC Res. Notes..

[270] Stall S (2019). Make scientific data FAIR. Nature.

[271] Tedersoo L (2021). Data sharing practices and data availability upon request differ across scientific disciplines. Sci. Data..

[272] de la Porte, B. & Higgs, R. Challenges in digitisation of cultural heritage material in the Western Cape, South Africa. *S. Afr. J. Inf. Manag.* **21**, a1104 (2019).

[273] Sayers EW (2022). Database resources of the National Center for Biotechnology Information in 2023. Nucl. Acids Res..

[274] Robertson T (2014). The GBIF integrated publishing toolkit: facilitating the efficient publishing of biodiversity data on the internet. PLoS One..

[275] Flemons, P., Guralnick, R., Krieger, J., Ranipeta, A. & Neufeld, D. A web-based GIS tool for exploring the world’s biodiversity: The Global Biodiversity Information Facility Mapping and Analysis Portal Application (GBIF-MAPA).*Ecol. Inform.* **2**, 49–60 (2007).

[276] Wieczorek J (2012). Darwin Core: an evolving community-developed biodiversity data standard. PLoS One..

[277] Borries, C. et al. Transparency, usability, and reproducibility: Guiding principles for improving comparative databases using primates as examples. *Evol. Anthropol.* **25**, 232–238 (2016). 10.1002/evan.21502 27753217

[278] Reed, D. et al. Digital data collection in paleoanthropology. *Evol. Anthropol.* **24**, 238–249 (2015). 10.1002/evan.21466 26662947

[279] Reed D, Harrison T, Kwekason A (2019). Eyasi Plateau Paleontological Expedition, Laetoli, Tanzania, fossil specimen database 1998-2005. Sci. Data..

[280] Reed, et al. In *New Geospatial Approaches to the Anthropological Sciences* (eds R. L. Anemone & G. C. Conroy) 211–224 (University of New Mexico Press., 2018).

[281] Reed DN (2023). Hominin nomenclature and the importance of information systems for managing complexity in paleoanthropology. J. Hum. Evol..

[282] Sithole, J. The challenges faced by African libraries and information centres in documenting and preserving indigenous knowledge. *IFLA J.* **33**, 117–123 (2007).

[283] Mabe, K. & Potgieter, A. Collaboration between libraries, archives and museums in South Africa. *S. Afr. J. Inf. Manag.* **23**, 1–8 (2021).

[284] de Jager EJ, Risser L, Mescam M, Fonta C, Beaudet A (2022). Sulci 3D mapping from human cranial endocasts: A powerful tool to study hominin brain evolution. Hum. Brain Mapp..

[285] Subsol, G., Gesquiere, G., Braga, J. & Thackeray, F. 3D automatic methods to segment “virtual” endocasts: state of the art and future directions. *Am. J. Phys. Anthropol.* **141**, 226–227 (2010).

[286] Milham, M. et al. Accelerating the evolution of nonhuman primate neuroimaging. *Neuron* **105**, 600–603 (2020). 10.1016/j.neuron.2019.12.023 PMC7610430 32078795

[287] Milham MP (2018). An Open Resource for Non-human Primate Imaging. Neuron.

[288] Messinger A (2021). A collaborative resource platform for non-human primate neuroimaging. Neuroimage.

[289] Heuer, K., Ghosh, S. S., Robinson Sterling, A. & Toro, R. Open Neuroimaging Laboratory. *Research Ideas and Outcomes* **2**, e9113 (2016).

[290] Heuer, K., Traut, N., de Sousa, A. A., Valk, S. & Toro, R. Diversity and evolution of cerebellar folding in mammals. *bioRxiv*, 2022.2012.2030.522292, 10.1101/2022.12.30.522292 (2023). 10.7554/eLife.85907 PMC10617990 37737580

[291] Clancy B, Darlington RB, Finlay BL (2001). Translating developmental time across mammalian species. Neuroscience.

[292] Marchant R, Tetard M, Pratiwi A, Adebayo M, de Garidel-Thoron T (2020). Automated analysis of foraminifera fossil records by image classification using a convolutional neural network. J. Micropalaeontol..

[293] Liu, X. et al. Automatic taxonomic identification based on the Fossil Image Dataset (>415,000 images) and deep convolutional neural networks. *Paleobiology* **49***,* 1–22 (2022).

[294] Greenwald NF (2022). Whole-cell segmentation of tissue images with human-level performance using large-scale data annotation and deep learning. Nat. Biotechnol..

[295] Sendhoff, B., Körner, E., Sporns, O., Ritter, H. & Doya, K. *Creating Brain-Like Intelligence*. Vol. 5436 (2009).

[296] Davies TG (2017). Open data and digital morphology. Proc. Biol. Sci..

[297] Rigby Dames BA (2023). Evolutionary and genomic perspectives of brain aging and neurodegenerative diseases. Prog. Brain Res..

[298] Khaitovich P (2008). Metabolic changes in schizophrenia and human brain evolution. Genome Biol..

[299] Griffa A, Van den Heuvel MP (2018). Rich-club neurocircuitry: function, evolution, and vulnerability. Dialogues Clin. Neurosci..

[300] Sousa AMM (2017). Molecular and cellular reorganization of neural circuits in the human lineage. Science.

[301] Benton ML (2021). The influence of evolutionary history on human health and disease. Nat. Rev. Genet..

[302] Calvey T (2019). Human self-domestication and the extended evolutionary synthesis of addiction: How humans evolved a unique vulnerability. Neuroscience.

[303] Jourjine N, Hoekstra HE (2021). Expanding evolutionary neuroscience: insights from comparing variation in behavior. Neuron.

[304] Calvey, T., de Sousa, A. A. & Beaudet, A. *From Fossils to Mind*. 1 edn, Vol. 275 (Elsevier, 2023). 10.1038/s42003-023-04803-4 PMC10262152 37311857

[305] Cohen KM, Finney SC, Gibbard PL, Fan JX (2013). The ICS International Chronostratigraphic Chart. Episodes.

[306] Benton, M. J. *Vertebrate Palaeontology*. 4 edn, 480 (Wiley Blackwell, 2014).

[307] Kemp, T. S. *The Origin and Evolution of Mammals*. (Oxford University Press, 2005).

[308] Sues, H.-D. *The Rise of Reptiles: 320 Million Years of Evolution*. (Johns Hopkins University Press, 2019).

[309] Kumar, S. et al. TimeTree 5: An Expanded Resource for Species Divergence Times. *Mol. Biol. Evol.* **39**, msac174 (2022). 10.1093/molbev/msac174 PMC9400175 35932227

[310] Wood, B., Doherty, D. & Boyle, E. In *Oxford Research Encyclopedia of Anthropology* (Oxford University Press, 2020).

[311] Lauters, P. *Valleculae on the frontal bone of a hadrosaurid dinosaur*, 10.5281/zenodo.7454914 (2023).

[312] Bardo, A. *Present day human hand grasping the same artifact by hand and hafted*, 10.5281/zenodo.7452329 (2023).

